# The Effects of 4 Weeks of Chiropractic Spinal Adjustments on Motor Function in People with Stroke: A Randomized Controlled Trial

**DOI:** 10.3390/brainsci11060676

**Published:** 2021-05-21

**Authors:** Kelly Holt, Imran Khan Niazi, Imran Amjad, Nitika Kumari, Usman Rashid, Jens Duehr, Muhammad Samran Navid, Muhammad Shafique, Heidi Haavik

**Affiliations:** 1 Centre for Chiropractic Research, New Zealand College of Chiropractic, Auckland 1060, New Zealand; kelly.holt@nzchiro.co.nz (K.H.); imran.amjad@nzchiro.co.nz (I.A.); Nitika.kumari@nzchiro.co.nz (N.K.); Jens.duehr@nzchiro.co.nz (J.D.); samran.navid@nzchiro.co.nz (M.S.N.); heidi.haavik@nzchiro.co.nz (H.H.); 2 Faculty of Health & Environmental Sciences, Health & Rehabilitation Research Institute, AUT University, Auckland 0627, New Zealand; usman.rashid@aut.ac.nz; 3 Department of Health Science and Technology, Aalborg University, 9220 Aalborg, Denmark; 4 Faculty of Rehabilitation and Allied Health Sciences and Department of Biomedical Engineering, Riphah International University, Islamabad 46000, Pakistan; muhammad.shafique@riphah.edu.pk

**Keywords:** stroke, motor function, chiropractic spinal adjustment, physical therapy, health-related quality of life, recovery of function

## Abstract

Chiropractic spinal adjustments have been shown to result in short-term increases in muscle strength in chronic stroke patients, however, the effect of longer-term chiropractic spinal adjustments on people with chronic stroke is unknown. This exploratory study assessed whether 4 weeks of chiropractic spinal adjustments, combined with physical therapy (chiro + PT), had a greater impact than sham chiropractic with physical therapy (sham + PT) did on motor function (Fugl Meyer Assessment, FMA) in 63 subacute or chronic stroke patients. Secondary outcomes included health-related quality of life and other measures of functional mobility and disability. Outcomes were assessed at baseline, 4 weeks (post-intervention), and 8 weeks (follow-up). Data were analyzed using linear mixed-effects models or generalized linear mixed models. A post-hoc responder analysis was performed to investigate the clinical significance of findings. At 4 weeks, there was a larger effect of chiro + PT, compared with sham + PT, on the FMA (difference = 6.1, *p* = 0.04). The responder analysis suggested the improvements in motor function seen following chiropractic spinal adjustments may have been clinically significant. There was also a robust improvement in both groups in most measures from baseline to the 4- and 8-week assessments, but between-group differences were no longer significant at the 8-week assessment. Four weeks of chiro + PT resulted in statistically significant improvements in motor function, compared with sham + PT, in people with subacute or chronic stroke. These improvements appear to be clinically important. Further trials, involving larger group sizes and longer follow-up and intervention periods, are required to corroborate these findings and further investigate the impacts of chiropractic spinal adjustments on motor function in post-stroke survivors. ClinicalTrials.gov Identifier NCT03849794.

## 1. Introduction

Stroke can result in persistent impairments of structure and function, which can lead to limitations of activity and a negative impact on quality of life [1,2]. Due to long term disability, many stroke survivors are dependent on their caregivers for assistance with activities of daily life, such as dressing, bathing, and toileting, which imposes an additional burden on society [3]. Regaining lost motor function is critical to post-stroke recovery [4]. Several rehabilitation protocols are available for motor recovery in people with stroke [5,6]; however, there is often a limit to the recovery that can be achieved using current rehabilitation approaches [7]. Therefore, if new techniques and approaches are identified that facilitate motor recovery following stroke, this will be beneficial for stroke survivors and society in general.

Chiropractic care has the potential to facilitate motor recovery in stroke survivors due to its influence on the central nervous system [8,9,10,11,12,13,14]. Chiropractic care constitutes a holistic approach to health, with a focus on the identification and correction of central segmental motor control (CSMC) problems that chiropractors often call vertebral subluxations [8,15,16,17]. CSMC problems are described as neurobiomechanical problems in the spine that cause ongoing maladaptive neuroplastic changes in the CNS [8,13,17]. CSMC problems are hypothesized to be caused by the stresses and strains of daily life, impacting central segmental motor control in the spine, which can result in a self-perpetuating motor control problem [16,17]. Various manual techniques, particularly high-velocity, low-amplitude adjustments (often called spinal manipulation), are applied to the subluxated spinal segments to correct the CSMC problems and facilitate the ability of the CNS to self-coordinate, self-regulate, adapt, and heal [16,17,18]. The spine is the biomechanical and neurological connection between the brain and limbs and there is evidence that changes in afferent signals from the spine can alter central neural processing [13], which can impact motor control of the limbs [11,12,14,19,20,21]. Numerous studies demonstrated that chiropractic spinal adjustments can alter paraspinal mechanical afferent input, as well as central somatosensory processing, sensorimotor integration, and motor control [13], not only relating to spinal motor control, but also of the limbs, pelvic floor muscles, and even jaw muscles [11,12,14,19,20,21,22,23]. Sensorimotor integration is the ability of the CNS to produce appropriate motor output by integrating sensory inputs from the body and environment [13,24,25,26]. It is essential for learning a new motor skill and re-learning lost motor skills following an injury [4]. Considering that people with chronic stroke have significant issues with sensorimotor integration and motor control [27], chiropractic spinal adjustments may have the potential to enhance neural plasticity, sensorimotor integration, and motor recovery following a stroke.

Previous studies demonstrated that a single session of chiropractic spinal adjustments can improve muscle strength in different populations [11,12,22,28]. Studies also revealed that a single session of chiropractic spinal adjustments can increase plantar flexor muscle strength in elite taekwondo athletes [12] and decrease quadriceps muscle inhibition, with an increase in quadriceps activation in people with anterior knee pain [20]. Niazi et al. [28] reported that a single session of chiropractic spinal adjustments improved plantar flexion strength in people with sub-clinical pain due to changes in spinal or supraspinal neural plasticity. A randomized controlled crossover trial of 12 individuals with chronic stroke found that a single session of chiropractic spinal adjustments enhanced plantar flexor muscle strength by an average of 64.2% [11]. Another study found that a single session of chiropractic spinal adjustments increased the amplitude of the N30 somatosensory evoked potential (SEP) peak (reflecting changes in early sensorimotor integration) in chronic stroke patients [9]. However, it is not known if any of these changes are functionally important, or whether longer-term chiropractic care can improve the functional ability, mobility, and quality of life in people with stroke.

When investigating whether chiropractic care may be beneficial to people with stroke, it must be taken into account that current interventions are already known to help with motor recovery in stroke survivors [5,6]. Therefore, they should not be withheld when studying a novel intervention. Hence, the primary objective of this exploratory study was to investigate the effects of 4 weeks of chiropractic care combined with usual physical therapy, compared with sham chiropractic combined with usual physical therapy, on motor function in people with chronic stroke. The secondary objectives of the study were to investigate the effects of 4 weeks of chiropractic care, combined with usual physical therapy care, on health-related quality of life, functional mobility, and dynamic balance, global disability, and functional lower limb strength in people with chronic stroke.

## 2. Methods

### 2.1. Design and Setting

The study was a parallel group, randomized controlled trial (RCT) and was conducted at the Rehabilitation Center of Railway General Hospital, Rawalpindi, Pakistan, from January to June 2019. The Ethical Review Committee of Riphah International University, Pakistan, approved the study (Riphah/RCRS/REC/000458). The study was registered with the U.S. National Institutes of Health ClinicalTrials.gov clinical trial registry (NCT03849794). Ethics approval: All procedures performed in studies involving human participants were in accordance with the ethical standards of the institutional and/or national research committee and with the 1964 Helsinki Declaration and its later amendments or comparable ethical standards. The study was approved by the Ethical Review Committee of Riphah International University, Pakistan (Riphah/RCRS/REC/000458).

### 2.2. Study Participants

Participants were patients from the Railway General Hospital database who had experienced a stroke at least 12 weeks prior to enrolment in the study, and had previously completed a rehabilitation program at the hospital. In this study, we refer to these participants as having subacute or chronic stroke, based on previously used classification criteria [29,30]. Potential participants were contacted by telephone and invited to participate in the study. All patients that agreed to participate and presented to Railway General Hospital during the study enrolment period were assessed for eligibility. Participants were included if they suffered from a stroke at least 12 weeks prior to their participation in the trial and scored less than 80 on a combined upper- and lower-extremity Fugl-Meyer Assessment (FMA) of motor function (i.e., they had significant motor impairment) [31,32]. Participants were excluded if they showed no evidence of spinal dysfunction (i.e., no presence of CSMC problem indicators, as identified by a chiropractor), had absolute contraindications to spinal adjustments (i.e., history of spinal fracture, atlantoaxial instability, spinal infection, spinal tumor, or cauda equina syndrome), or previously had an adverse response to chiropractic spinal adjustments or spinal manipulation. Written consent was obtained from all volunteers before they participated in the study.

### 2.3. Interventions

The interventions were 4 weeks of chiropractic plus physical therapy (chiro + PT) and 4 weeks of sham chiropractic plus physical therapy (sham + PT). A standalone chiropractic intervention was not considered in this exploratory study as it would have meant withholding an intervention that is known to be effective in order to test a novel intervention [6].

#### 2.3.1. Chiropractic Intervention

In the chiro + PT group, New Zealand registered chiropractors checked participants for CSMC problems and adjusted them, where necessary, during the intervention period. Participants were checked by the chiropractor approximately three times per week for 4 weeks. Clinical indicators for CSMC problems included tenderness to palpation, restricted intersegmental motion, asymmetric muscle tension, and blocked joint-play or end-feel. These clinical indicators are routinely used by chiropractors when analyzing the spine and have previously been shown to be reliable for the identification of CSMC problems when used as a multidimensional battery of tests [33,34]. Chiropractic adjustments were provided where clinically warranted and were either manual, high-velocity, low-amplitude thrusts, or instrument-assisted thrusts to the spine or pelvic joints [35]. Multiple levels of the spine were adjusted in each participant, if deemed appropriate, based on the chiropractic examination. Each chiropractic visit lasted approximately 15 min. No other interventions were provided by the chiropractor.

#### 2.3.2. Sham Chiropractic Intervention

Blinding of participants in a trial involving a physical intervention is challenging due to the manual nature of the intervention [36,37]. One advantage of doing this study in Pakistan is that chiropractic is relatively unknown in the country [38]. In a recent survey of university students in Lahore, Pakistan, including pharmacy students, more than two-thirds of respondents were unaware that chiropractic care involved spinal manipulation and that it is used as a treatment for low back pain [38]. This lack of knowledge about chiropractic provides a unique opportunity to study its effects with the enhanced potential of successful participant blinding. In order to reduce the impact of contextual effects on study outcomes, the control group received a sham chiropractic intervention.

Participants in the sham + PT control group saw the same chiropractors, at the same frequency, as those in the experimental group. The chiropractor performed the same assessment for CSMC problems as the experimental group and chiropractic visits were of roughly the same duration as those in the experimental group. However, instead of applying manual or instrument-assisted thrusts to the spine, the chiropractor either positioned participants as if they were going to thrust on the spine, but did not provide a manual thrust, or they placed an adjusting instrument, set to the minimum setting, lateral to the spine or on the chiropractor’s hand or arm and produced a clicking sound with the instrument. Communication between the chiropractor and participants was very limited in both groups due to language barriers, so translators were used to ask participants to move into the required positions for the control and experimental procedures. To test the effectiveness of participant blinding, following the 4-week intervention period, participants in both groups were asked to indicate, using a yes or no response, whether they thought they had received active chiropractic care.

#### 2.3.3. Physical Therapy Intervention

Both groups underwent three comprehensive sessions of physical therapy per week with an estimated duration of 40 min each. For both groups, the physical therapy program consisted of muscle stretching (such as shoulder adductor; internal rotator; elbow, wrist, and hip flexor; hip adductor; knee muscle; and plantar flexor stretches), strengthening (for weak groups of muscles in upper and lower limbs), balance exercises in sitting and standing positions, sit-to-stand practice, transfer practice according to patient needs, walking, stair climbing, upper limb functional training (reach, grasp, and hand-to-mouth activities), muscle tone inhibition techniques, postural stability control, sensory techniques, and daily functional activities. Hot pack and TENS were used to reduce pain or for muscle relaxation if required [39]. Furthermore, the participants were encouraged to continue performing exercises at home. The physical therapist that delivered the physical therapy treatment had ten years of experience treating patients with neurological disorders.

### 2.4. Outcome Measures

All outcome measures were assessed at baseline, 4 weeks (post-intervention), and 8 weeks (to assess retention effects). The primary outcome measure was the Fugl-Meyer Assessment for motor function for the combined upper and lower limbs, and the primary endpoint was the 4-week assessment. Secondary outcome measures included the Stroke Specific Quality of Life scale, the Timed Up and Go test, the Modified Rankin Scale, and the five-repetition Sit-to-Stand Test.

Potential harm or adverse events were investigated by asking the physical therapists and translators assisting the chiropractors to ask participants, at scheduled intervention visits, about any injuries or perceived adverse effects of care that may have occurred during the trial.

#### 2.4.1. Fugl-Meyer Assessment Scale

The Fugl-Meyer Assessment scale (FMA) is a highly reliable and valid performance-based impairment scale that can measure recovery after stroke [32,40,41,42]. It is recommended as one of the core measures for the evaluation of stroke recovery [43]. It can assess motor function, balance, sensation, and pain. For the present study, motor function was used as the primary outcome measure. The maximum score for motor function for the upper extremity (FMA-UE) is 66, and for the lower extremity (FMA-LE) is 34. Patients with a score of 0 to 35 are said to have a severe impairment, 36 to 55 is moderately severe, 56 to 79 is moderate, and 80 or greater is mild [44]. To be included in the current study, participants were required to have an FMA score of less than 80. The motor assessment includes an examination of the shoulder, elbow, forearm, wrist, and hand in the upper limbs, and the hip, knee, and ankle in the lower limbs. The examination includes an assessment of movement ability, coordination, speed, and control, as well as an assessment of reflex activity [32,44].

#### 2.4.2. Stroke Specific Quality of Life Scale

The Stroke Specific Quality Of Life scale (SS-QOL) is considered a reliable and valid tool for the assessment of quality of life after stroke [45,46]. The SS-QOL has 49 items with 12 domains: energy, family roles, language, mobility, mood, personality, self-care, social roles, thinking, upper-extremity function, vision, and work/productivity. The response is measured using a 5-point Likert scale in which higher scores indicate better function [45].

#### 2.4.3. Timed up and Go Test

The Timed Up and Go (TUG) is considered a reliable and valid instrument to evaluate the functional mobility of people with stroke [47,48]. In this test, participants were asked to stand up, walk for three meters, turn, walk back, and sit down. The time taken to complete this task was recorded using a stopwatch [47].

#### 2.4.4. Modified Rankin Scale

The modified Rankin Scale (mRS) is used as a measure of participation and global disability in the stroke population [49]. It scores the participants from 0 (no symptoms at all) to +5 (severe disability).

#### 2.4.5. Five-Repetition Sit-to-Stand Test

The Sit-to-Stand Test (SST) is a reliable and valid test to assess functional lower limb muscle strength [50]. It has several variations, such as time taken to perform a given number of SST or the maximum number of SST performed in 30 or 60 s. For the present study, the five-repetition SST (5SST) test was utilized, as it measures physical performance in frail elderly [51] and has been previously used as an outcome measure to determine the effects of different interventions on individuals with total hip and knee arthroplasty, vestibular dysfunction, and stroke [52,53,54]. Participants were instructed to perform the sit-to-stand activity five times without using their hands. The time taken to complete the five-repetitions was measured using a stopwatch. A chair without armrests was used and the mean of three trials was recorded for analysis. There was one-minute rest time after each trial.

### 2.5. Randomization and Blinding

Randomization was carried out following the baseline assessment using an online minimization tool (QMinim, Telethon Kids Institute, Perth, Australia) [55]. Age, gender, and FMA score at baseline were used as an input for minimization. All participants, the outcomes assessors, and the physical therapists providing the physical therapy intervention were blinded to group allocation. The statistician responsible for analysis of the data was also blinded to group allocation, as all recorded data were anonymized and coded before being provided. The chiropractors providing chiropractic care could not be blinded to group allocation.

### 2.6. Statistical Analysis

As an exploratory trial, pre-planned sample size calculations were not made, as predictions of effect sizes could not be made based on relevant previous research. Instead, it was decided to use a recruitment window of 3 months and enroll as many participants as possible during this time period. The goal was to enroll 100 participants, if possible, during this timeframe. Three months was the maximum recruitment period possible, based on the availability of chiropractors to provide the chiropractic intervention in Pakistan.

The primary null hypothesis for the analysis was that there was no difference in motor function, measured with the FMA, between the chiro + PT and sham + PT groups. Pre-specified secondary null hypotheses stated that there were no differences between the two groups in FMA-UE, FMA-LE, SS-QOL, TUG, mRS, and 5SST.

To test these hypotheses, data collected during the study was collated in an Excel (Microsoft Corp., Redmond, WA, USA) spreadsheet with groups relabeled for the purpose of blinding. Statistical analyses were conducted in R version 4.0.0 using the packages lme4 and robustlmm [56,57,58]. A detailed report of the statistical analysis is available in the supplementary file. For FMA, FMA-UE, FMA-LE, SS-QOL, TUG, mRS, and 5SST; separate mixed models were set up for longitudinal analysis of covariance. Each model included the pre-randomization baseline score as a covariate to adjust for potential baseline differences [59,60], had a saturated fixed-effects structure consisting of group, time (a factor indicating 4 weeks and 8 weeks), their interaction, and a random intercept effect for participants. For 5SST, analysis of covariance was not possible as a linear relationship between baseline and post-intervention scores was not evident in the raw data. Thus, an analysis of variance was performed by including baseline as an additional time-point with the remaining model structure consistent with models for the other outcomes. To correct for baseline differences in 5SST, baseline means were subtracted while calculating the means at the post-intervention time-points. For TUG and 5SST, a robust linear mixed model was set up to cater for outliers. For mRS, a generalized linear mixed model with Gamma family and identity link was setup to model mean response [61]. For the remaining outcomes, linear mixed models were used.

A post-hoc responder analysis was performed to investigate the potential clinical significance of between-group differences in the FMA. To compare the efficacy of two active interventions in a clinical trial it is recommended that the proportion of participants in each group that meet the minimum clinically important difference (MCID) should be calculated and compared between groups [62]. It is considered to be inappropriate to compare mean differences to known MCIDs as the MCID is a metric that should be based on longitudinal differences in individual patients [62]. MCIDs have not previously been calculated for a combined FMA score in people with chronic stroke, so the proportion of participants in each group that surpassed relevant MCIDs for the FMA in either the upper or lower limb was used to compare group differences, as these are known [63,64]. This was felt to be the most pragmatic approach since the primary outcome measure was the combined FMA score. A cut-off of 6 for the FMA-LE was used and both 4.25 and 7.25 were used in separate analyses for the FMA-UE [63,64]. Two values were used for the FMA-UE evaluation, as this is the range of MCIDs that relate to five different upper limb functional anchors that were established in a similar cohort of stroke survivors [63]. Chi-squared tests were performed to assess whether responder proportions statistically differed between groups. Based on the proportions of participants in each group displaying clinically meaningful improvements, the number needed to treat (NNT) was calculated, as it can be argued that this is a more clinically relevant method for evaluating differences between different intervention strategies [62].

Effect sizes for between-group and within-group differences are reported with standard errors and 95% confidence intervals that were obtained from the models. P-values for testing the primary and secondary null hypotheses were obtained with z- or t-tests based on these estimates. The statistical significance level was set at 0.05.

## 3. Results

**Figure 1:**
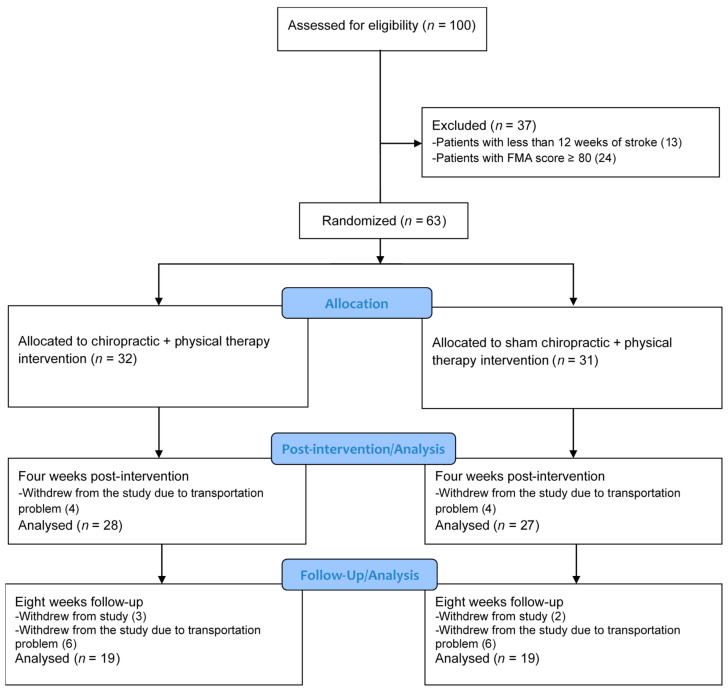
CONSORT study flow diagram.

One-hundred volunteers were assessed for eligibility between January and March 2019, and 63 met the inclusion criteria and agreed to participate in the trial. Fifty-five participants completed the 4-week assessment (*n* = 28 in the chiro + PT group, *n* = 27 in the sham + PT group) and 38 completed the 8-week assessment (*n* = 19 in each group). The participants who dropped out during the first 4 weeks of the study (*n* = 8) all had issues with caregiver availability or transportation limitations to the hospital where the study was taking place. There was a considerable loss to follow-up between the 4- and 8-week assessments as many study participants had traveled from surrounding regions with their caregivers and could only stay away from home for the time that they were receiving active intervention. No adverse events or reports of harm were received during the trial. The study flow is given in Figure 1. Participant’s clinical characteristics are given in Table 1.

### 3.1. Between-Group Differences

**Figure 2:**
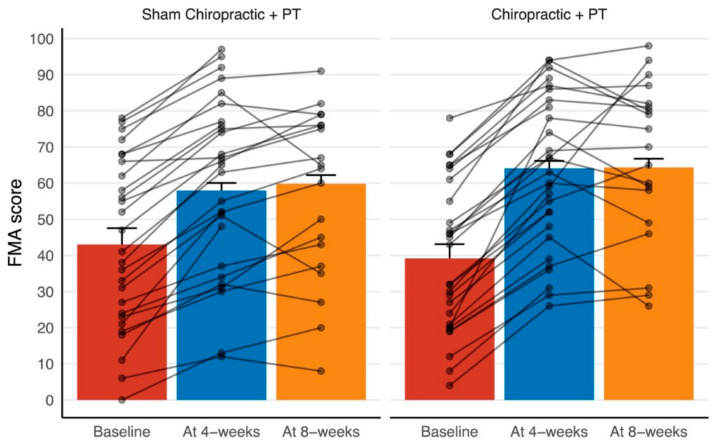
Participant-wise FMA scores, along with group-wise means and standard errors for the two interventions at pre-randomization baseline, at the end of the interventions (4 weeks), and the follow-up (8 weeks). Note: FMA stands for Fugl-Meyer Assessment and PT stands for physical therapy. The baseline means and standard errors are calculated from the raw data, whereas the remaining statistics are from the fitted model, which computes means and standard errors after adjusting for the baseline scores. Similar illustrations of remaining outcomes are available in the supplementary file.

The participant-wise raw data for the FMA score are shown in Figure 2. This figure suggests a consistent improvement in the FMA score at the end of the 4-week intervention period across the two groups. The majority of the participants remained at a similarly improved level at the 8-week follow-up, with some continuing to show additional improvements and some declining in score.

The differences between chiro + PT and sham + PT in the primary and secondary outcome measures are given in Table 2. These results show a larger effect of chiro + PT compared with sham + PT on the combined FMA (between-group difference = 6.1, *p* = 0.04) and FMA-LE (between-group difference = 2.9, *p* = 0.02) scales at the 4-week follow-up. The remaining differences are statistically non-significant.

Since there was a statistically significant between-group difference in the primary outcome (combined FMA) at the primary endpoint, a post-hoc responder analysis was performed (see Table 3). This analysis was intended to give an indication of the clinical significance of the observed results. Between-group differences for the proportion of participants displaying MCIDs in FMA (either UE or LE) were calculated using the MCID cut-off scores previously mentioned, and results are presented in Table 3 [63,64]. At the 4-week primary endpoint, almost all participants (98%) improved based on the lower FMA-UE MCID (4.25), so this responder analysis was somewhat meaningless. However, when using the higher (7.25) FMA-UE MCID score, combined with the FMA-LE cut-off of 6, there was a significant between-group difference (chiro + PT = 96%, sham + PT = 78%, *X*^2^ (1, *N* = 55) = 4.3, *p* = 0.04). The NNT is 5 when calculated using these latter cut-off scores at four weeks.

### 3.2. Within-Group Differences

The estimated means of the outcome measures at 4 weeks and at 8 weeks for the two intervention groups are given in Table 4. These estimates and the accompanying hypothesis tests suggest that there was a robust (*p* < 0.0001) increase in FMA, FMA-UE, FMA-LE, and SS-QOL across both the groups from baseline to post-intervention (at 4 weeks) and that this increase was maintained above the baseline at the 8-week follow-up.

For TUG, a decrease below the baseline was only significant at 4 weeks in the chiro + PT group (*p* = 0.047). The mRS decreased and stayed below the baseline across both groups, whereas the improvement in 5SST compared with the baseline was not significant for either group.

### 3.3. Participant Blinding

After the 4-week intervention period, all participants who completed the trial were asked if they thought they had received active chiropractic care. Twenty-six of the 28 participants in the chiro + PT group and 26 of the 27 participants in the sham + PT group believed they had received active chiropractic care. This suggests that participant blinding was successful, with 95% of participants across the two groups believing they had received active chiropractic care, with no between-group differences present.

## 4. Discussion

This study is the first multi-session study to evaluate the effects of chiropractic spinal adjustments on motor function in stroke survivors. The combination of chiropractic spinal adjustments and physical therapy improved motor function, particularly lower limb motor function, after 4 weeks of care, compared with sham chiropractic spinal adjustments plus physical therapy. The improvements in motor function in the chiropractic group compared with the sham group were no longer significant at the 8-week follow-up. This may have been due to a diminishing effect of the chiropractic care over the 4-week follow-up period with no ongoing care. It is also possible that high participant drop-out between the 4- and 8-week assessments resulted in a type II error. There were no significant between-group improvements in SS-QOL, TUG, mRS, or 5SST scores at the 4- or 8-week assessments. However, there were significant within-group improvements in FMA, FMA-UE, FMA-LE, SS-QOL, and mRS scores across time for both groups. A significant within-group decrease in the TUG score was noted in the chiro + PT group at 4 weeks, indicating an improvement in functional mobility and dynamic balance. There were no significant within-group changes in 5SST at any time point.

There is strong evidence that physical therapy facilitates motor recovery in people with stroke [65,66]. Four weeks of usual physical therapy care has been shown to increase the mean FMA-UE score from 14.30 (2.20) at baseline to 22.05 (3.12) at one-month follow-up testing in people with acute stroke [67]. A combination of different physical therapy approaches has been found to be more effective in facilitating lower-limb motor function recovery in people with stroke [68]. In the present study, combining chiropractic care with usual physical therapy care further enhanced the beneficial effects of physical therapy on motor function recovery in people with subacute or chronic stroke.

Of interest is that the chiropractic care that was provided in the present study did not involve any therapeutic or rehabilitative interventions directed at the impaired limbs and it did not directly seek to treat the symptoms that were associated with stroke. Instead, the chiropractors aimed to locate and correct areas of spinal dysfunction, or CSMC problems [15,17], as this is thought to improve the central integration of somatosensory input from the spine and body [13]. CSMC problems can be reliably detected in relatively healthy populations [33], and a broad range of people in the community seek chiropractic care for wellness care or a range of conditions and issues [69,70,71,72,73,74,75,76]. Therefore, the chiropractic care in the present study did not markedly differ from the care that a chiropractor may provide to a patient who had not suffered from a stroke. Further clinical trials should investigate the effects of chiropractic care on sensorimotor function in other populations that may benefit from enhanced sensorimotor control.

Determining whether the between-group improvements in the current study were clinically meaningful is not clear-cut. To establish whether there is a clinically important difference between the two interventions that are effective can be challenging [62]. It is not as simple as comparing improvements in one group to the improvements in the other group to see whether they exceed a known MCID. This evaluation should be based on changes in individual subjects, as opposed to group means. Therefore it has been recommended that, when comparing the efficacy of two interventions in a clinical trial, a responder analysis should be used [62]. In the present study, the responder analysis suggested that chiropractic spinal adjustments resulted in clinically meaningful improvements in motor function when added to physical therapy care for chronic stroke survivors at the primary endpoint when using 7.25 as the MCID for the FMA-UE (chiro + PT = 96% vs. sham + PT = 78%, *p* = 0.04). The NNT calculated from the responder analysis was 5, which suggests that if five chronic stroke patients received chiropractic care, in conjunction with physical therapy, one will achieve additional favorable outcomes. A single-digit value for NNT generally denotes a worthwhile difference when comparing interventions [77]. An NNT of 5 compares favorably with studies included in a systematic review of strength training in stroke recovery [78] and suggests that the addition of chiropractic care to the physical therapy program was clinically important. However, this post-hoc analysis should be interpreted with caution, as significance depended on the magnitude of MCID and the time-point used [78]. In future studies, to better identify between-group differences and make firmer conclusions about the clinical significance of chiropractic care for enhancing motor function in stroke survivors, studies should be larger, have a longer-term follow-up, and should consider including a more homogenous stroke population (e.g., either significant upper limb deficit or lower limb deficit, as opposed to a combination of both.) Predefined MCIDs should also be used, with higher MCIDs more likely to result in a meaningful responder analysis if an active control is used.

The improvement in motor function following the addition of 4 weeks of chiropractic care to usual physical therapy care supports the previous findings of our recent basic science randomized controlled crossover studies in people with chronic stroke [9,11]. A change in SEP peak amplitude (reflecting early sensorimotor integration) [9] and plantar flexor muscle strength, with an increase in cortical drive [11], was found after a single session of chiropractic spinal adjustments in people with chronic stroke. These findings suggest chiropractic spinal adjustments modulate cortical function [79,80,81] by influencing somatosensory processing, sensorimotor integration, and motor control [8,13,82]. These changes have been hypothesized to be due do altered spinal sensory input and altered central neural processing following the correction of CSMC problems [8,83,84]. Numerous studies have shown that spinal function impacts proprioception and motor control of the limbs and that chiropractic spinal adjustments can result in improvements in proprioception and muscle strength in both the upper and lower limbs [12,14,21,26,28,84,85,86], which may be important for motor recovery in people with stroke. Recovery, or re-learning, of lost motor function after an injury requires effective somatosensory processing and sensorimotor integration [4]. Therefore, the ability of chiropractic spinal adjustments to impact these mechanisms and produce long-lasting alterations in central neural function may explain the improvement in motor function seen in the present study.

Despite the improved motor function in the intervention group compared with the control group in the present study, and the fact that the follow-up scores were higher than the baseline in both groups, the groups were not statistically different at the 8-week follow-up. This finding is likely related to the large drop-out rate (31%), which resulted in a smaller sample that undermined the statistical power of the study. Future studies should take this potential drop-out rate into account in sample size calculations.

Improvement in motor function following the addition of chiropractic care to physical therapy did not result in statistically significant between-group improvements in activity and participation-based scales, such as SS-QOL, TUG, mRS, and 5SST. This may have been due to the small sample size, and type II errors cannot be ruled out in the evaluation of secondary outcome measures. A within-group improvement in health-related quality of life, global disability, functional mobility, and dynamic balance were observed in both groups across time. It must be noted that the above improvements were seen in people with chronic stroke, where the average time since stroke was 30 weeks or 7.5 months. Numerous studies have reported a plateau in “recovery potential” with time elapsed since stroke [87,88]. Therefore, an important question remains regarding the potential effects of chiropractic care if provided closer to stroke onset.

The improvement in health-related quality of life over time in both groups may be related to the increase in motor function, functional mobility, and dynamic balance reflected in the improved FMA and TUG scores. Recovery of the upper limb and lower limb sensorimotor function and balance have been found to predict the quality of life in a stroke population [89]. Recently, Martino Cinnera et al. [90] found that lower limb motor recovery affected the quality of life more than motor recovery of the upper limb following two months of stroke rehabilitation. Chiropractic care has also been shown to have a positive influence on the quality of life in different populations [91,92,93]. The present study is the first study to report within-group positive effects of chiropractic care, combined with physical therapy, on health-related quality of life in people with stroke. This supports further investigation of the effects of chiropractic care on quality of life in stroke and other populations in clinical trials.

### Strengths and Limitations

Statistically significant between-group improvements were observed in the full FMA and FMA for the lower limb at 4 weeks with a sample size of 55. This suggests that the study was adequately powered for the primary outcome measure at the primary endpoint. However, we recognize that this sample size may not have been large enough to detect between-group changes in the secondary outcomes measured. Therefore, type II errors may have occurred. As an exploratory study, we utilized a variety of outcome measures and made multiple comparisons without making adjustments to *p*-values. This is a limitation as it increases the chances of making type I errors, but is considered to be appropriate when exploring new areas of research such as this [94]. Future large scale RCTs targeting activity and participation level outcome measures can utilize the estimates of this study for sample size calculations. Future research should utilize longer intervention and follow-up periods and attempt to establish the optimal frequency of chiropractic care in people with stroke. Lastly, regardless of group allocation, more than 95% of the participants in the present study believed that they had undergone an active chiropractic intervention, which indicates adequate blinding of the participants. This is difficult to achieve in trials involving manual intervention [37,95] and suggests that between-group differences were not due to contextual or placebo effects. This is a strength of the study.

## 5. Conclusions

Improvements in motor function were observed when chiropractic care was added to 4 weeks of physical therapy care in people with subacute or chronic stroke. These improvements were statistically significant and a post-hoc responder analysis suggested they were also likely to be clinically significant. Chiropractic spinal adjustments may therefore be beneficial for people with motor impairments associated with subacute or chronic stroke. Further research, involving larger group sizes and longer-term follow-up and intervention periods, is required to corroborate these findings and further investigate the impacts of chiropractic care on motor function in people with stroke.

## Figures and Tables

**Table 1:** Clinical characteristics of participants in each group.

Variables	Chiro + PT	Sham + PT
**Gender**		
Male (n)	18	16
Female (n)	10	11
Age, years (mean +/− SD)	53.3 +/− 14.0	58.5 +/− 11.3
**Side of body affected by stroke**		
Left (n)	14	12
Right (n)	14	15
Time since stroke, months (mean +/− SD)	30.0 +/− 36.6	27.3 +/− 31.5
Subacute stage (12–24 weeks since stroke, n)	5	4
Chronic stage (>24 weeks since stroke, n)	23	23
**Type of stroke**		
Ischemic (n)	24	25
Hemorrhagic (n)	4	2

N, number of participants; SD, standard deviation.

**Table 2:** Difference between groups in the primary and secondary outcome measures.

Outcome	Time	Mean Difference ± SE [95% CI]	*p*-Value
**Primary:**			
FMA	4 weeks	6.1 ± 2.9 [0.4, 11.9]	0.04 *
	8 weeks	4.5 ± 3.4 [−2.2, 11.2]	0.2
**Secondary:**			
FMA-UE	4 weeks	2.9 ± 2.5 [−2.0, 7.9]	0.2
	8 weeks	3.0 ± 2.9 [−2.8, 8.8]	0.3
FMA-LE	4 weeks	2.9 ± 1.2 [0.5, 5.3]	0.02 *
	8 weeks	1.9 ± 1.4 [−0.9, 4.6]	0.2
SS-QOL	4 weeks	−9.2 ± 10.4 [−30.0, 11.5]	0.4
	8 weeks	16.7 ± 11.5 [−6.2, 39.6]	0.2
TUG	4 weeks	−1.2 ± 2.2 [−5.5, 3.2]	0.6
	8 weeks	0.9 ± 2.7 [−4.4, 6.2]	0.7
mRS	4 weeks	0.0 ± 0.3 [−0.5, 0.5]	0.9
	8 weeks	0.1 ± 0.3 [−0.4, 0.6]	0.7
STS	4 weeks	−1.5 ± 4.2 [−9.6, 6.6]	0.7
	8 weeks	1.0 ± 4.6 [−8.0, 9.9]	0.8

Mean difference = chiro + PT − sham + PT, * denotes the null hypothesis is rejected. These marginal mean differences were estimated by the statistical models by holding the baseline scores constant across the two groups, except in the case of 5SST for which adjustment was done by subtracting baseline means from the follow-up means. For 5SST, mean difference is defined as [chiro + PT − (chiro + PT) Baseline] − [sham + PT − (sham + PT) Baseline]. FMA, Fugal-Meyer Assessment; UE, upper extremity; LE, lower extremity; SS-QOL, Stroke Specific Quality of Life; TUG, Time Up and Go; mRS, Modified Rankin Scale; STS, Sit-to-Stand Test.

**Table 3:** Difference between groups in proportion of responders in the FMA.

Outcome	Time	Chiro + PT Responder % [n Responders/n Total]	Sham + PT Responder % [n Responders/n Total]	*p*-Value
FMA UE, and/or LE Responder (MCID cut-off UE = 4.25, LE cut-off = 6)	4 weeks	100% [28/28]	96% [26/27]	0.3
	8 weeks	63% [12/19]	37% [7/19]	0.1
FMA UE, and/or LE Responder (MCID cut-off UE = 7.25, LE cut-off = 6)	4 weeks	96% [27/28]	78% [21/27]	0.04 *
	8 weeks	84% [16/19]	79% [15/19]	0.7

FMA, Fugl-Meyer Assessment; UE, upper extremity; LE, lower extremity; MCID, minimum clinically important difference.

**Table 4:** Estimated within-group marginal mean differences for primary and secondary outcome measures.

Outcome	Time	Baseline Mean	Mean ± SE, *p*-Value	
			Chiropractic + PT	Sham + PT
**Primary:**				
FMA	At 4 weeks	40.9	64.1 ± 2.0, <0.001 *	58.0 ± 2.1, <0.001 *
	At 8 weeks		64.3 ± 2.4, <0.001 *	59.8 ± 2.4, <0.001 *
**Secondary:**				
FMA-UE	At 4 weeks	24.1	38.9 ± 1.7, <0.001 *	36.0 ± 1.8, <0.001 *
	At 8 weeks		40.3 ± 2.1, <0.001 *	37.3 ± 2.1, <0.001 *
FMA-LE	At 4 weeks	16.8	25.0 ± 0.8, <0.001 *	22.1 ± 0.9, <0.001 *
	At 8 weeks		24.2 ± 1.0, <0.001 *	22.4 ± 1.0, <0.001 *
SS-QOL	At 4 weeks	122.1	162.5 ± 7.2, <0.001 *	171.7 ± 7.4, <0.001 *
	At 8 weeks		190.6 ± 8.1, <0.001 *	174.0 ± 8.1, <0.001 *
TUG	At 4 weeks	23.7	20.5 ± 1.6, 0.047 *	21.7 ± 1.5, 0.2
	At 8 weeks		21.3 ± 1.8, 0.2	20.4 ± 2.0, 0.1
mRS	At 4 weeks	2.8	2.4 ± 0.2, 0.01 *	2.4 ± 0.2, 0.02 *
	At 8 weeks		2.2 ± 0.2, <0.001 *	2.1 ± 0.2, <0.001 *
5SST	Baseline		M_B_: 21.3 ± 2.1	M_B_: 22.7 ± 2.0
	At 4 weeks		17.9 ± 2.1, 0.3	20.8 ± 2.0, 0.5
	At 8 weeks		17.4 ± 2.3, 0.2	17.8 ± 2.6, 0.1

* denotes the null hypothesis is rejected. These marginal means were estimated by the statistical models by holding the baseline scores constant across the two groups, except in the case of 5SST, for which baseline means were also estimated by the respective model. Confidence intervals are presented in the supplementary file. FMA, Fugal-Meyer Assessment; UE, upper extremity; LE, lower extremity; SS-QOL, Stroke Specific Quality of Life; TUG, Time Up and Go; mRS, Modified Rankin Scale; STS, Sit-to-Stand Test.

## Data Availability

Reasonable requests for data can be made to the corresponding author but ethics committee approval will need to be granted prior to sharing any data.

## Supplementary Materials

The following are available online at https://www.mdpi.com/article/10.3390/brainsci11060676/s1, Statistics Report: The effects of 4 weeks of chiropractic spinal adjustment on motor function in people with stroke: A randomized controlled trial.
