# Supplementary material for: The Effects of 4 Weeks of Chiropractic Spinal Adjustments on Motor Function in People with Stroke: A Randomized Controlled Trial
Source: Brain Sci. 2021 May 21;11(6):676. doi: 10.3390/brainsci11060676 (PMC8224305; doi:10.3390/brainsci11060676)
Supplement: Supplementary file 1 [file brainsci-11-00676-s001.zip › brainsci-1173977-supplementary.pdf]

Statistics Report: The effects of 4 weeks of chiropractic spinal adjustment on motor function in people with stroke: A randomized controlled trial

Usman Rashid

Contents

1 Data Visualisations **2**
1.1 Fugl-Meyer Assessment Scale . . . 2
1.2 Fugl-Meyer Assessment Scale – Upper Extremity . . . 3
1.3 Fugl-Meyer Assessment Scale – Lower Extremity . . . 4
1.4 Stroke Specific Quality of Life Scale (QOL) . . . 5
1.5 Timed Up and Go Test (TUG) . . . 6
1.6 Modified Rankin Scale (mRS) . . . 7
1.7 Five-repetition Sit-to-Stand Test (SST) . . . 8
1.8 Baseline versus Follow-up . . . 9
1.8.1 Fugl-Meyer Assessment Scale – Full, Upper Extremity, Lower Extremity . . . 9
1.8.2 QOL, TUG, mRS, SST . . . 10

2 Statistical Models **11**
2.1 Longitudnal Analysis of Covariance . . . 11
2.2 Analysis of Variance . . . 11
2.3 Diagnostics for the Models . . . 12
2.3.1 Fugl-Meyer Assessment Scale . . . 12
2.3.2 Fugl-Meyer Assessment Scale – Upper Extremity . . . 13
2.3.3 Fugl-Meyer Assessment Scale – Lower Extremity . . . 14
2.3.4 QOL . . . 15
2.3.5 TUG . . . 16
2.3.6 SST . . . 18

3 Results **20**
3.1 Fugl-Meyer Assessment Scale . . . 20
3.2 Fugl-Meyer Assessment Scale – Upper Extremity . . . 20
3.3 Fugl-Meyer Assessment Scale – Lower Extremity . . . 20
3.4 QOL . . . 21
3.5 TUG . . . 21
3.6 mRS . . . 21
3.7 SST . . . 22

1 Data Visualisations

Participant-wise scores along with group-wise means and standard errors for the two interventions at pre-randomization baseline, at the end of the interventions (4-weeks) and at the follow-up (8-weeks). The baseline means and standard errors are calculated from the raw data, whereas the remaining statistics are from the fitted models which compute means and standard errors after adjusting for the baseline scores. For SST, baseline means are also estimated by the respective model and, thus, baseline adjustment is not possible.

1.1 Fugl-Meyer Assessment Scale

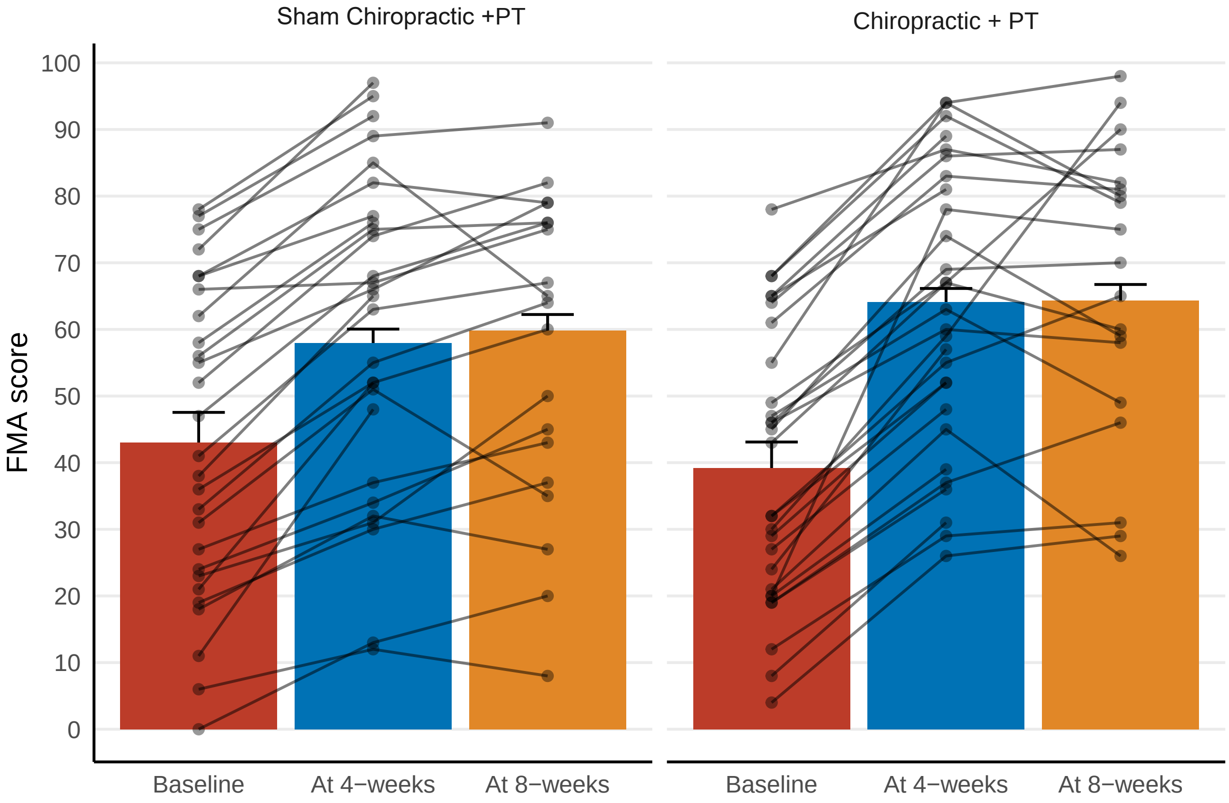

1.2 Fugl-Meyer Assessment Scale – Upper Extremity

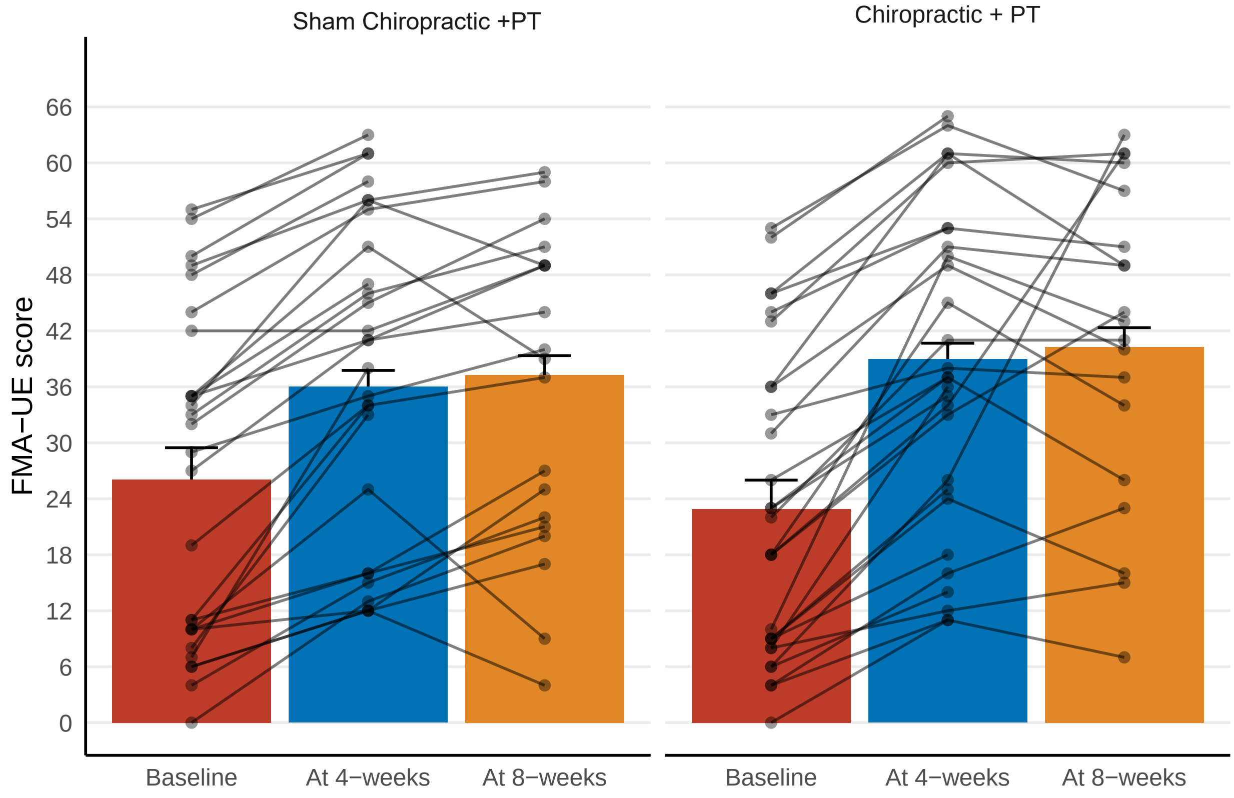

1.3 Fugl-Meyer Assessment Scale – Lower Extremity

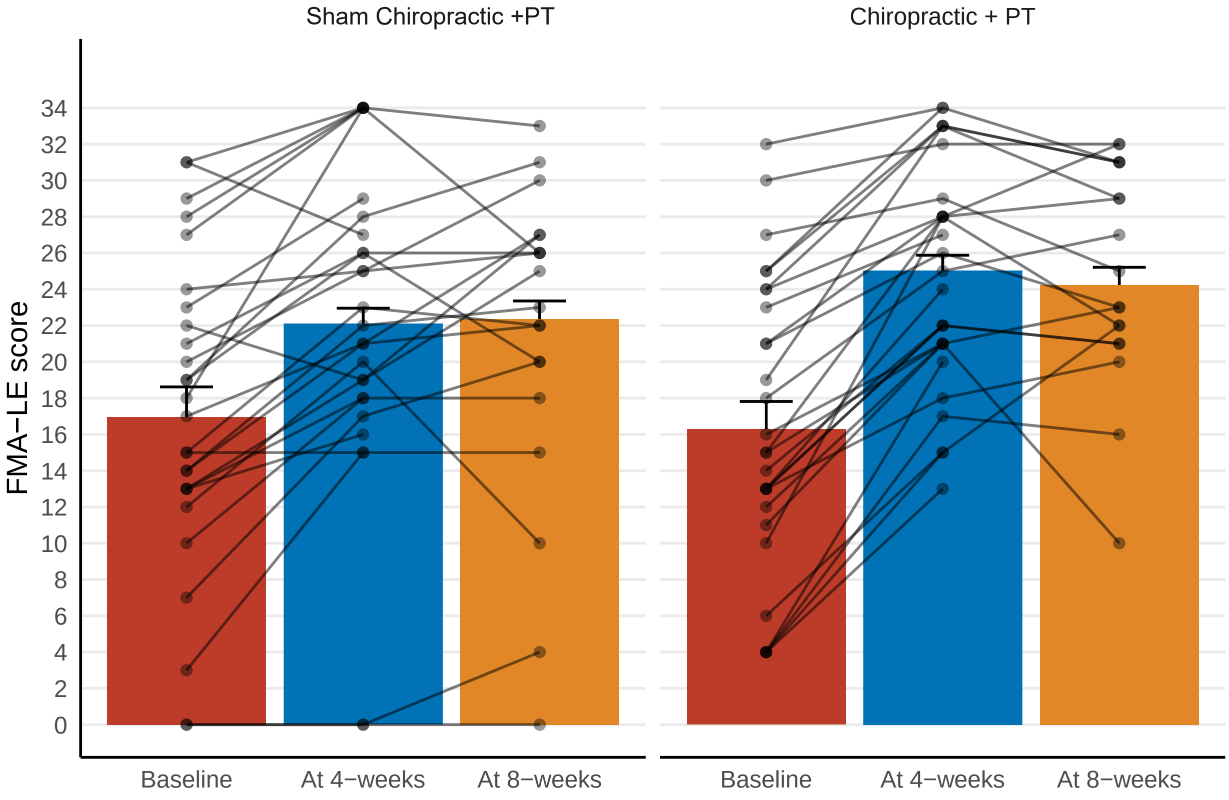

1.4 Stroke Specific Quality of Life Scale (QOL)

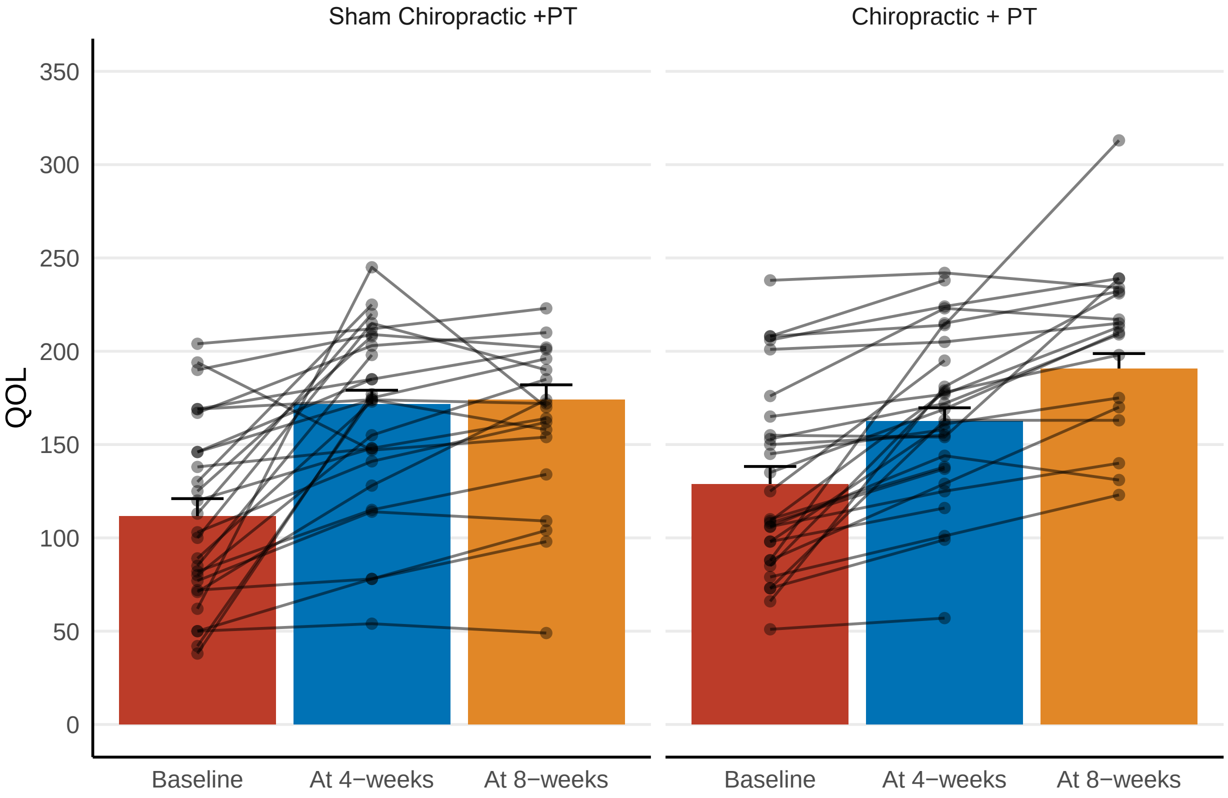

1.5 Timed Up and Go Test (TUG)

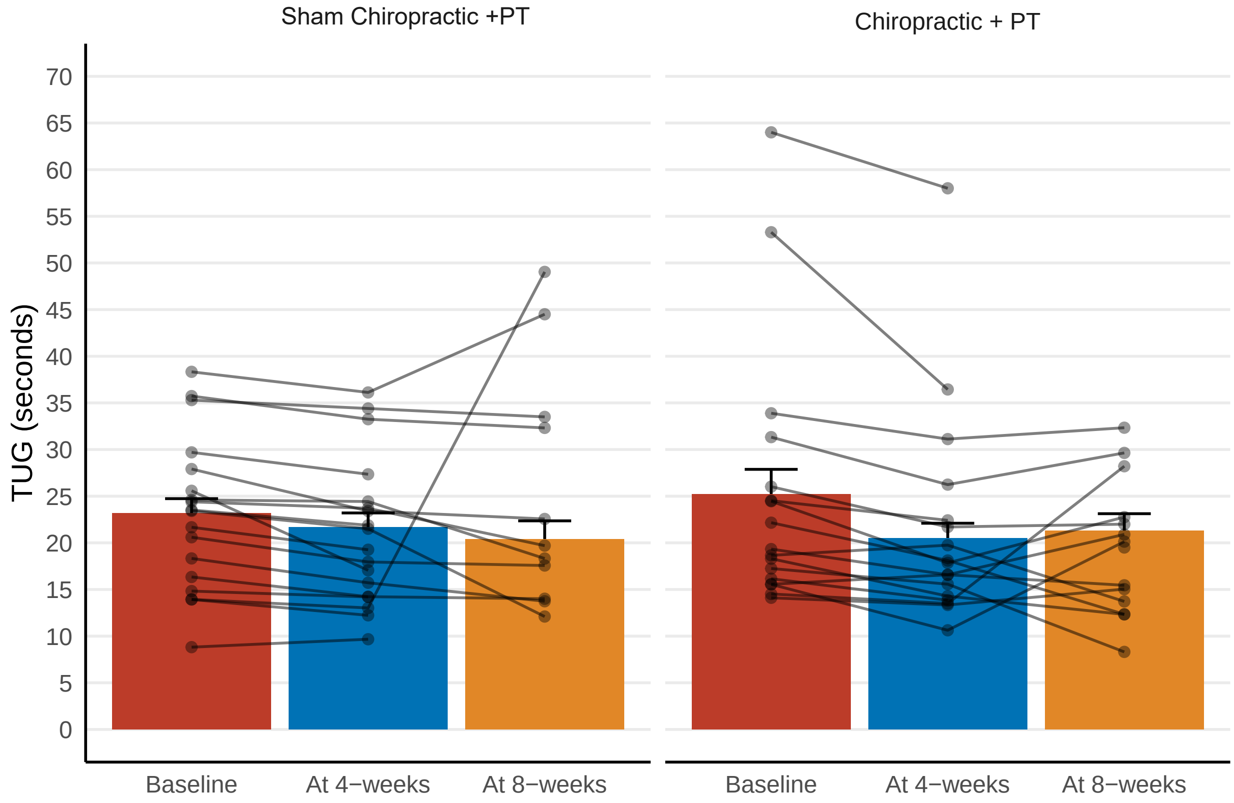

1.6 Modified Rankin Scale (mRS)

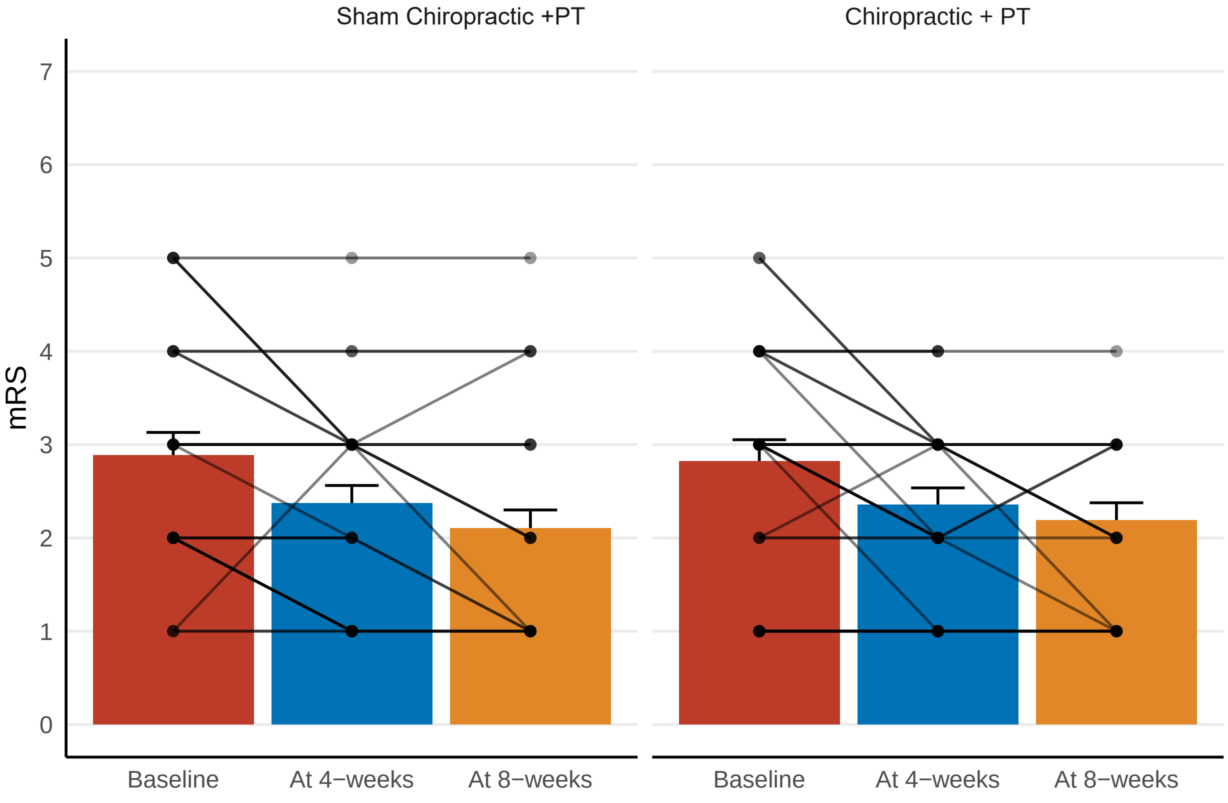

1.7 Five-repetition Sit-to-Stand Test (SST)

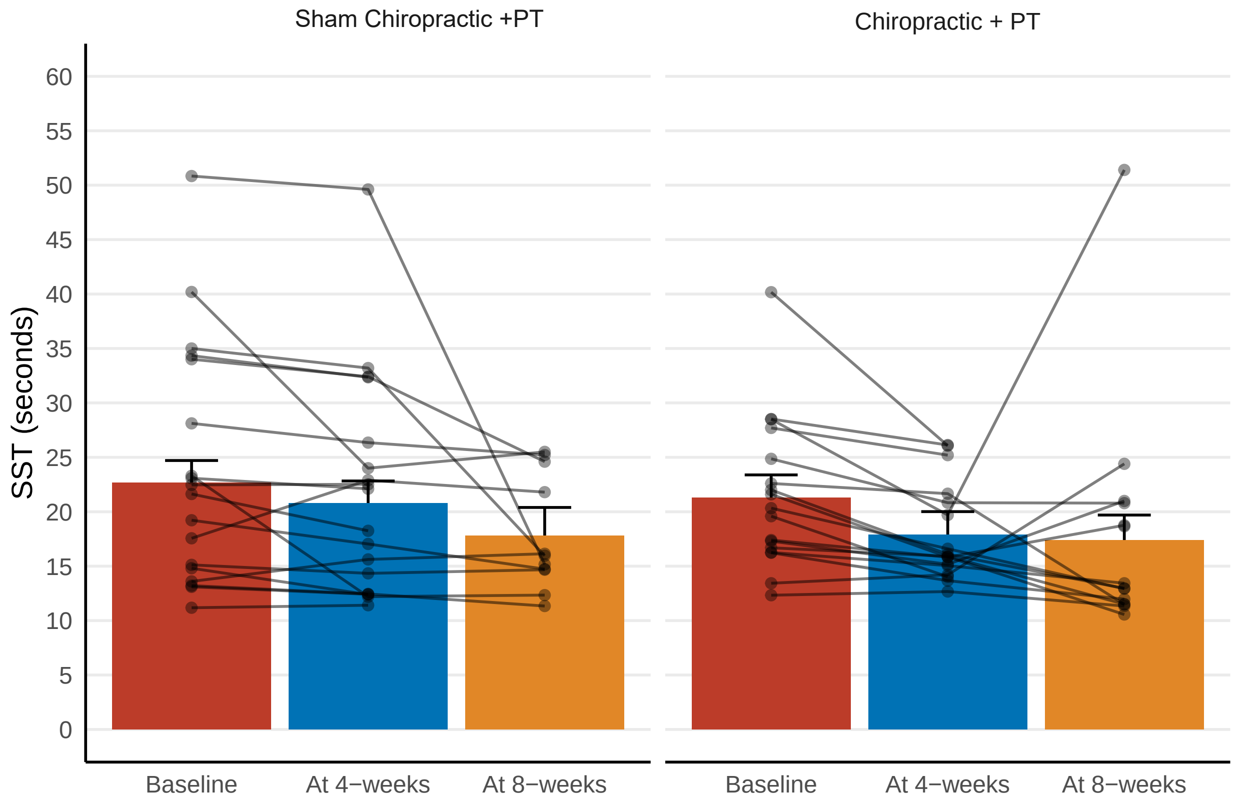

1.8 Baseline versus Follow-up

1.8.1 Fugl-Meyer Assessment Scale – Full, Upper Extremity, Lower Extremity

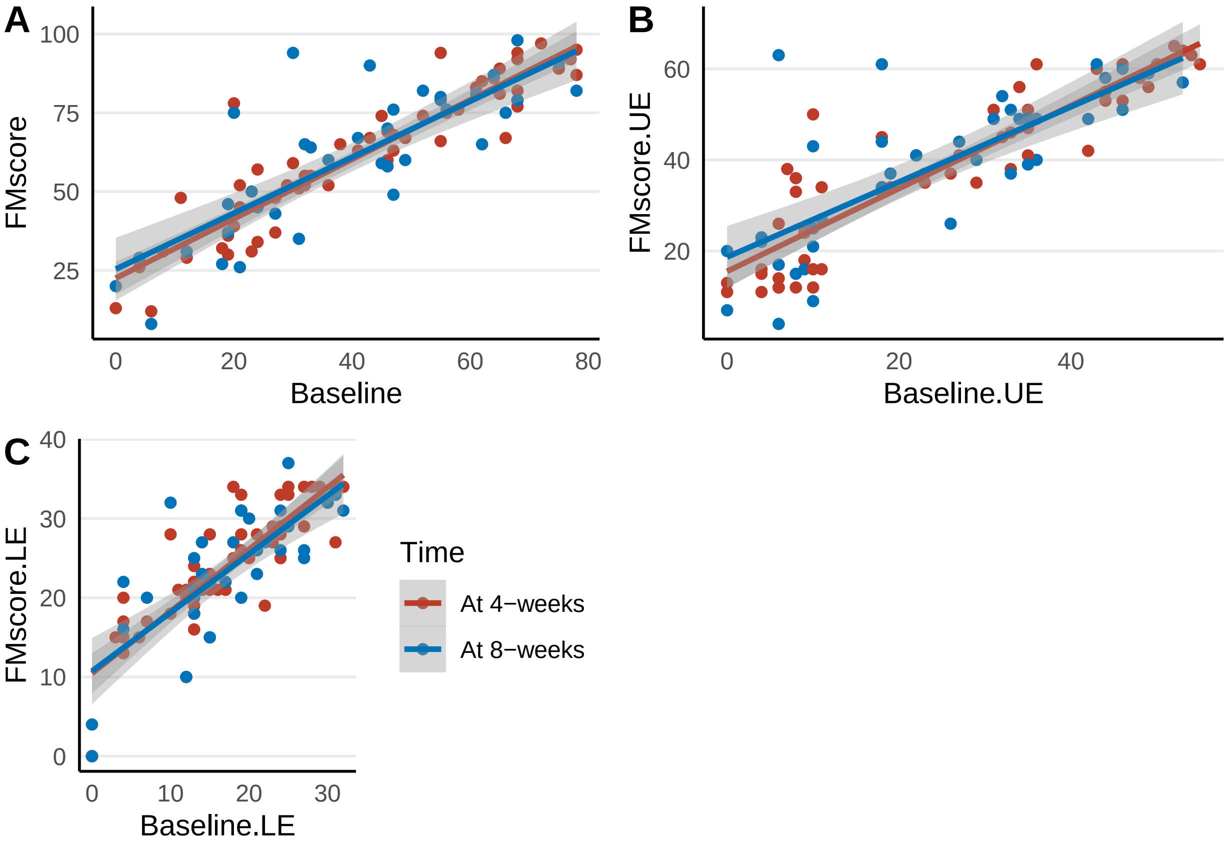

1.8.2 QOL, TUG, mRS, SST

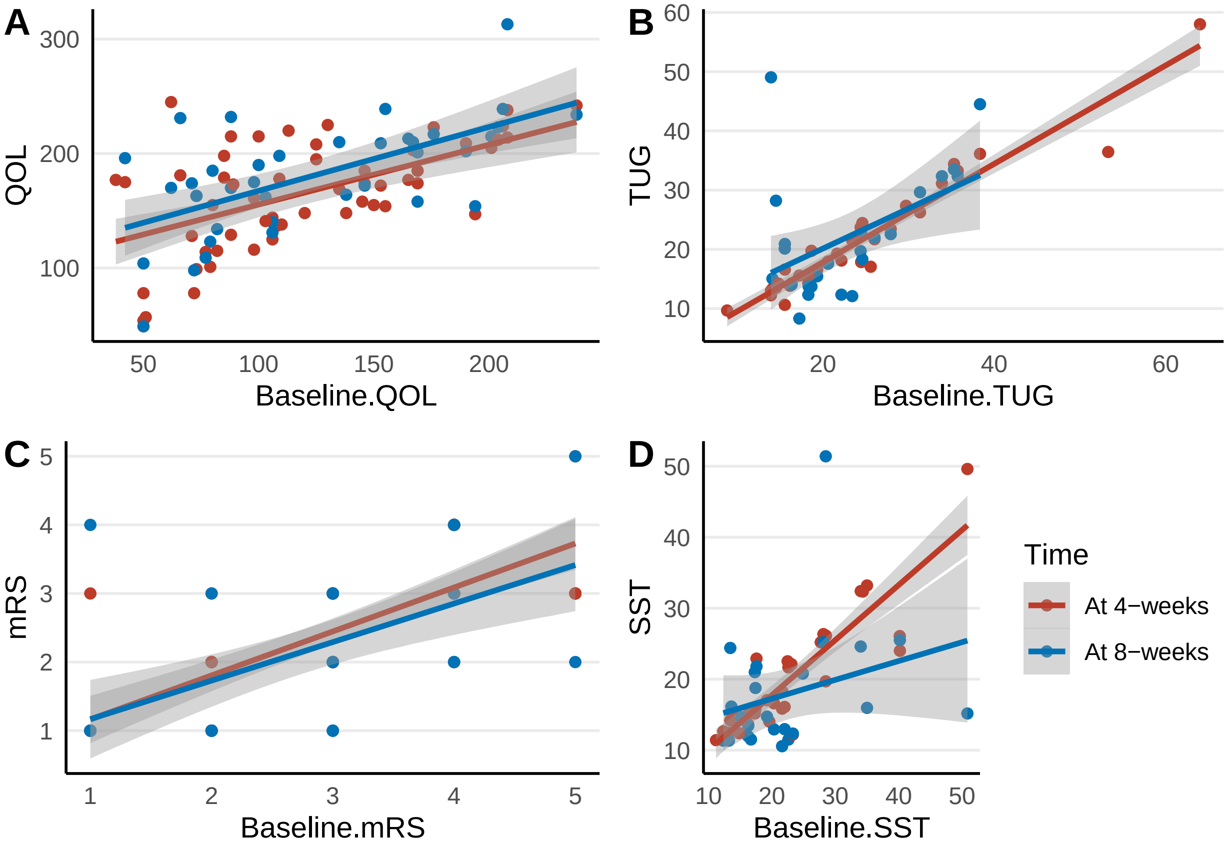

2 Statistical Models

2.1 Longitudnal Analysis of Covariance

```
lmerModel.full  <- lmer(FMscore ~ Baseline + Group*Time + (1|PartId),
                        Datasource,
                        na.action = na.omit)

lmerModel.UE    <- lmer(FMscore.UE ~ Baseline.UE + Group*Time + (1|PartId),
                        Datasource.UE,
                        na.action = na.omit)

lmerModel.LE    <- lmer(FMscore.LE ~ Baseline.LE + Group*Time + (1|PartId),
                        Datasource.LE,
                        na.action = na.omit)

lmerModel.QOL   <- lmer(QOL ~ Baseline.QOL + Group*Time + (1|PartId),
                        Datasource.QOL,
                        na.action = na.omit)

rlmerModel.TUG  <- rlmer(TUG ~ Baseline.TUG + Group*Time + (1|PartId),
                         Datasource.TUG,
                         na.action = na.omit)

glmerModel.mRS  <- glmer(mRS ~ Baseline.mRS + Group*Time + (1|PartId),
                         Datasource.mRS,
                         na.action = na.omit,
                         family = Gamma(link = "identity"))
```

2.2 Analysis of Variance

```
rlmerModel.SST  <- rlmer(SST ~ Group*Time + (1|PartId),
                         Datasource.SST.including.baseline,
                         na.action = na.omit)
```

2.3 Diagnostics for the Models

2.3.1 Fugl-Meyer Assessment Scale

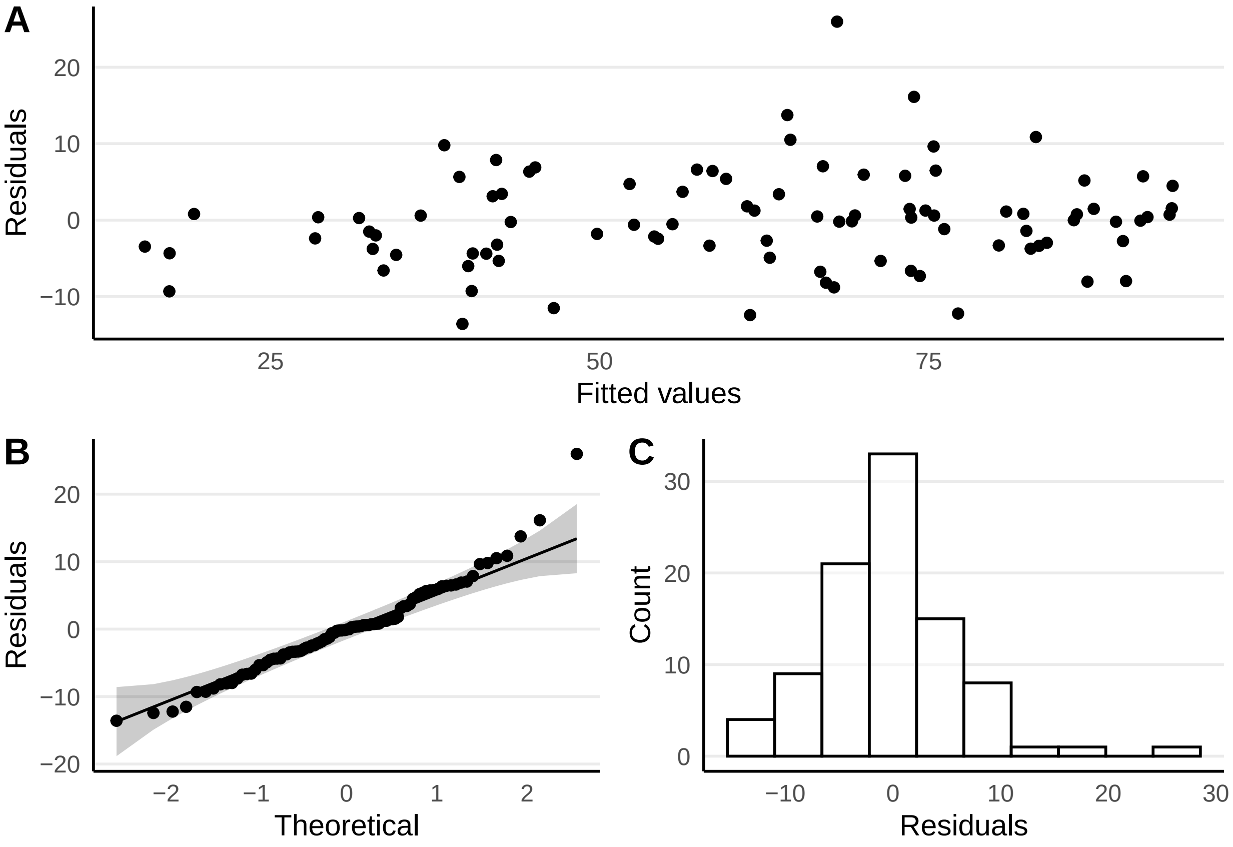

2.3.2 Fugl-Meyer Assessment Scale – Upper Extremity

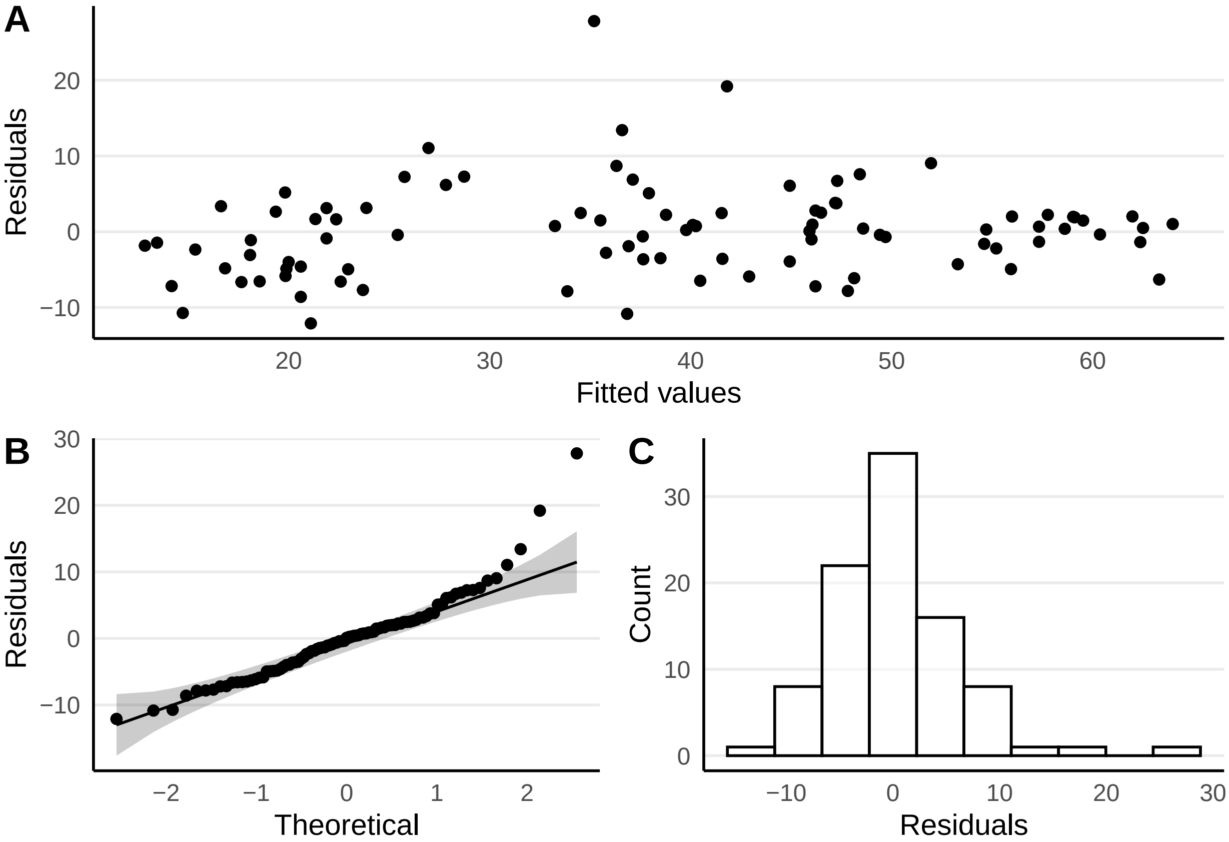

2.3.3 Fugl-Meyer Assessment Scale – Lower Extremity

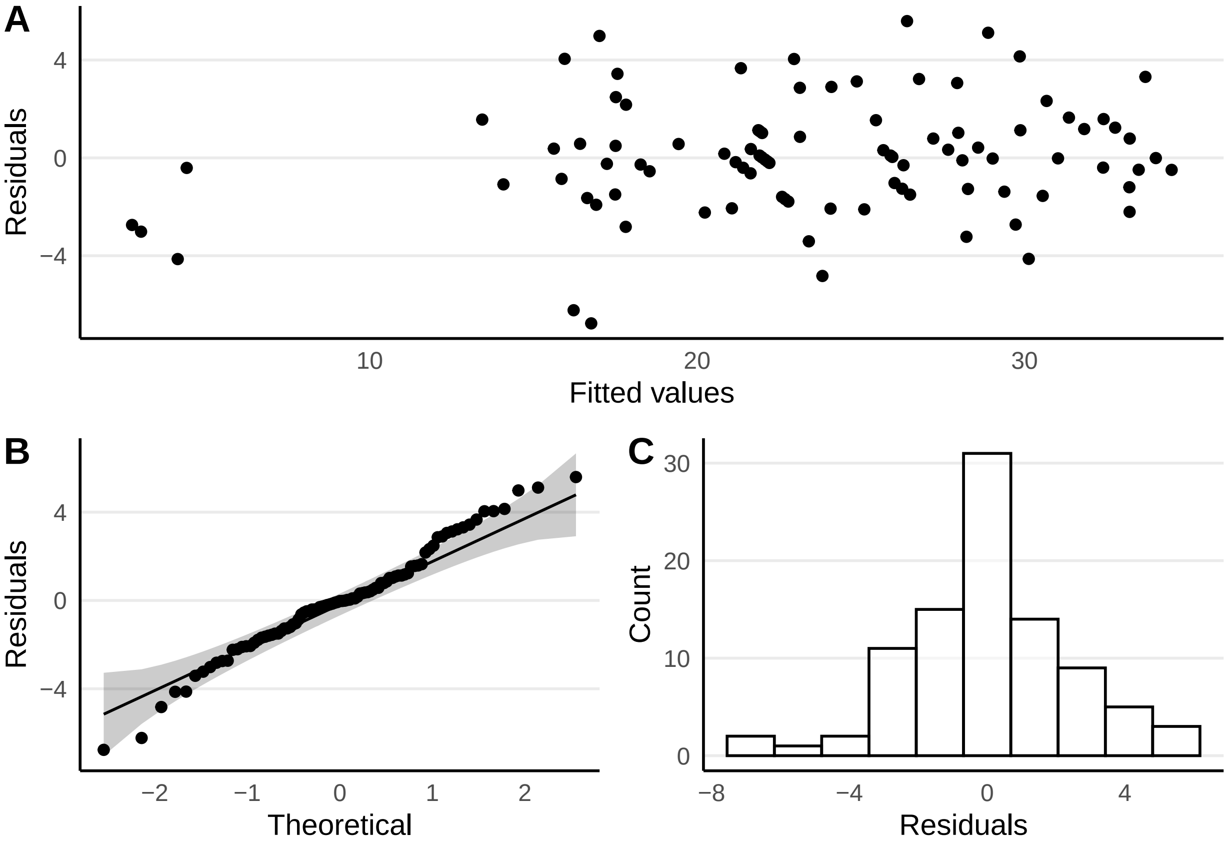

2.3.4 QOL

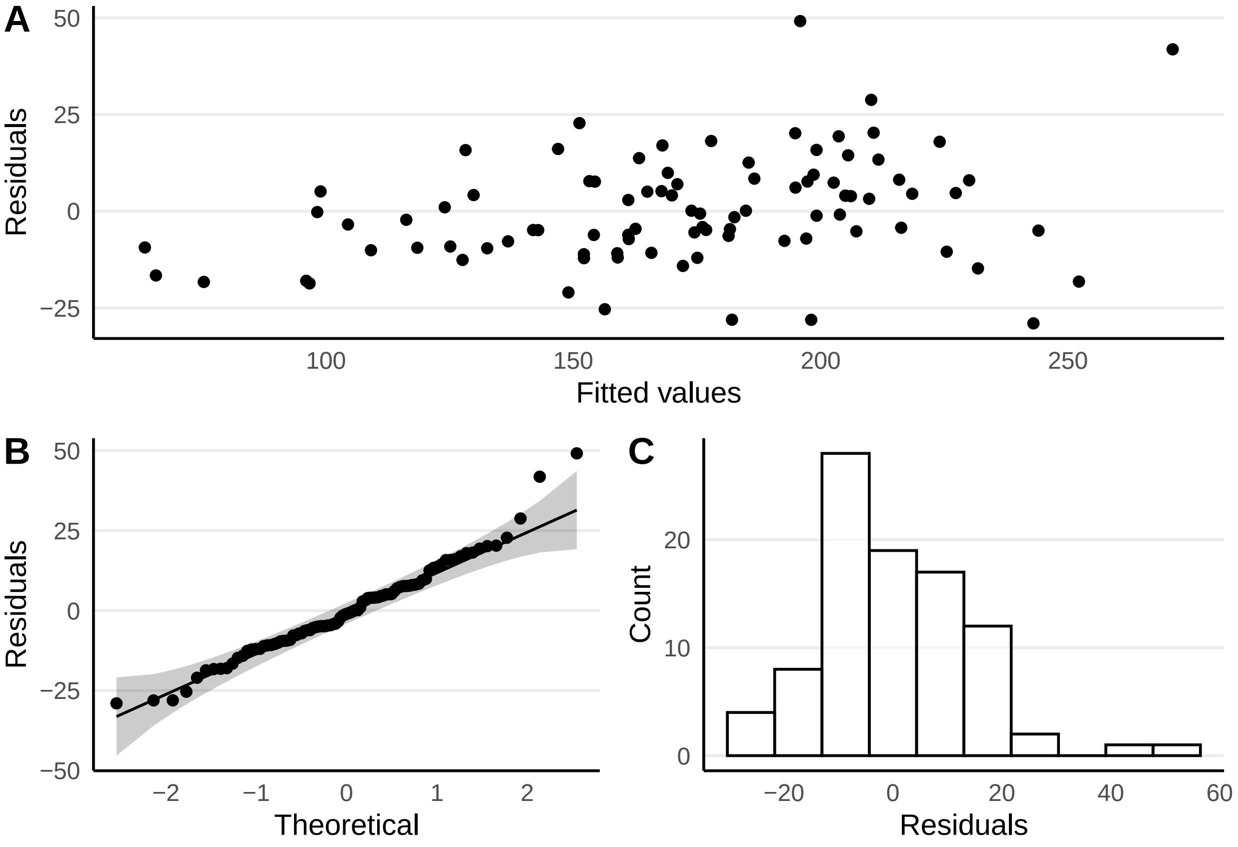
A
Residuals
Fitted values
50
25
0
−25
100
150
200
250
B
Residuals
Theoretical
50
25
0
−25
−50
−2
−1
0
1
2
C
Count
Residuals
20
10
0
−20
0
20
40
60

2.3.5 TUG

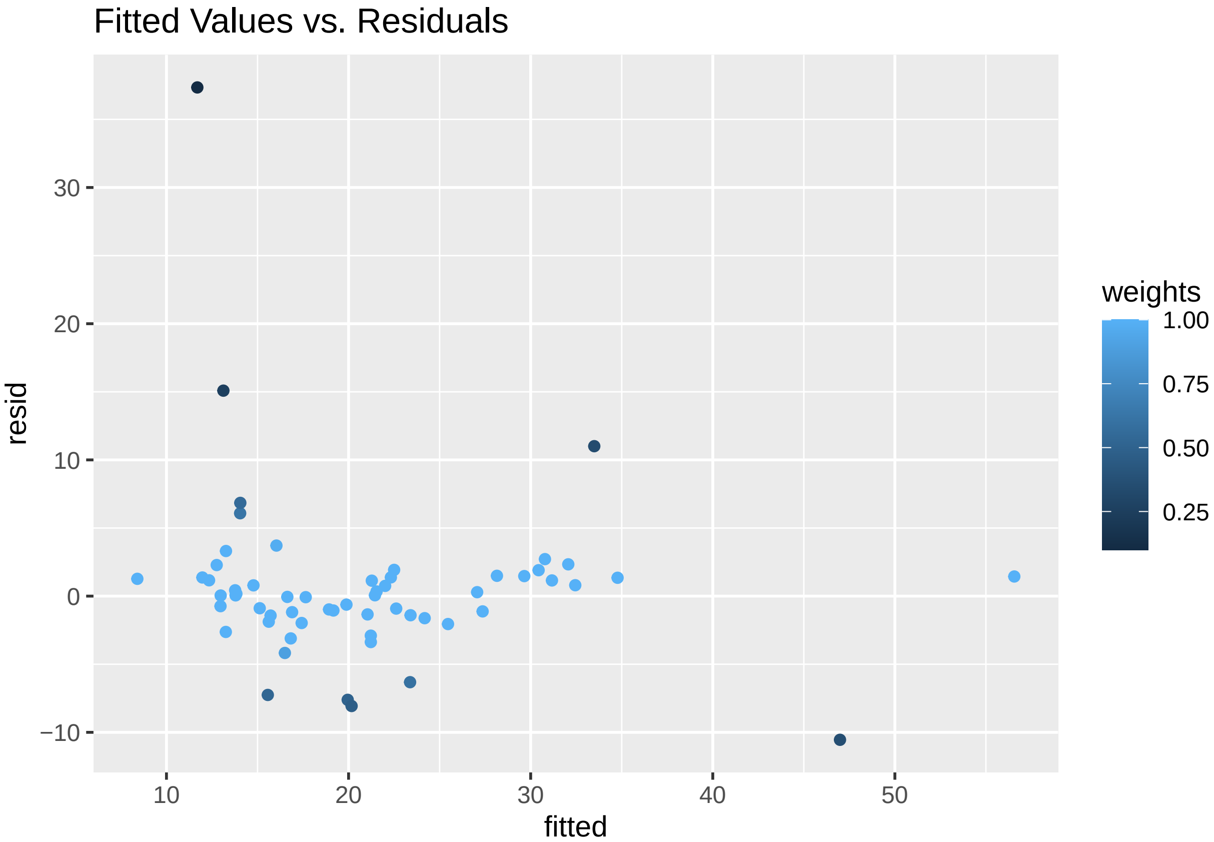
Fitted Values vs. Residuals
resid
fitted
30
20
10
0
−10
10
20
30
40
50
weights
1.00
0.75
0.50
0.25

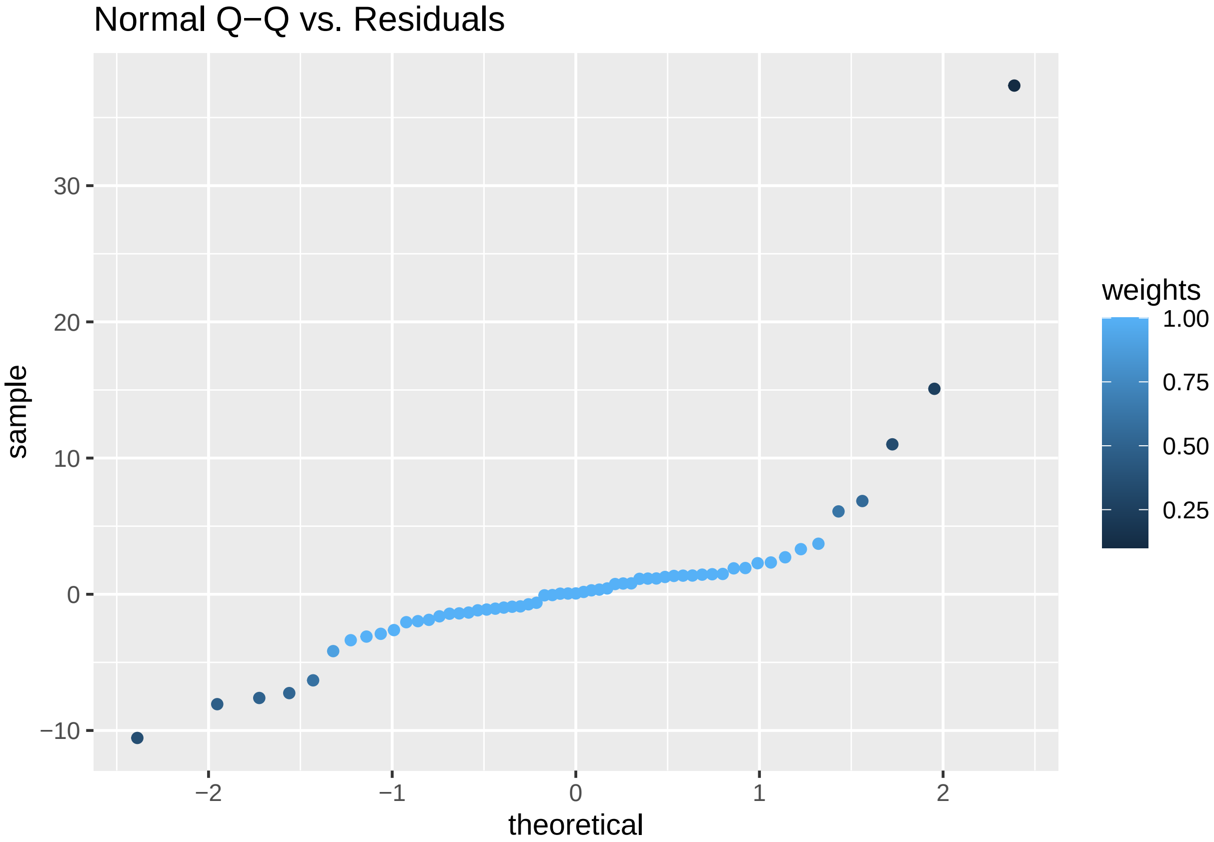
Normal Q−Q vs. Residuals
sample
theoretical
30
20
10
0
−10
−2
−1
0
1
2
weights
1.00
0.75
0.50
0.25

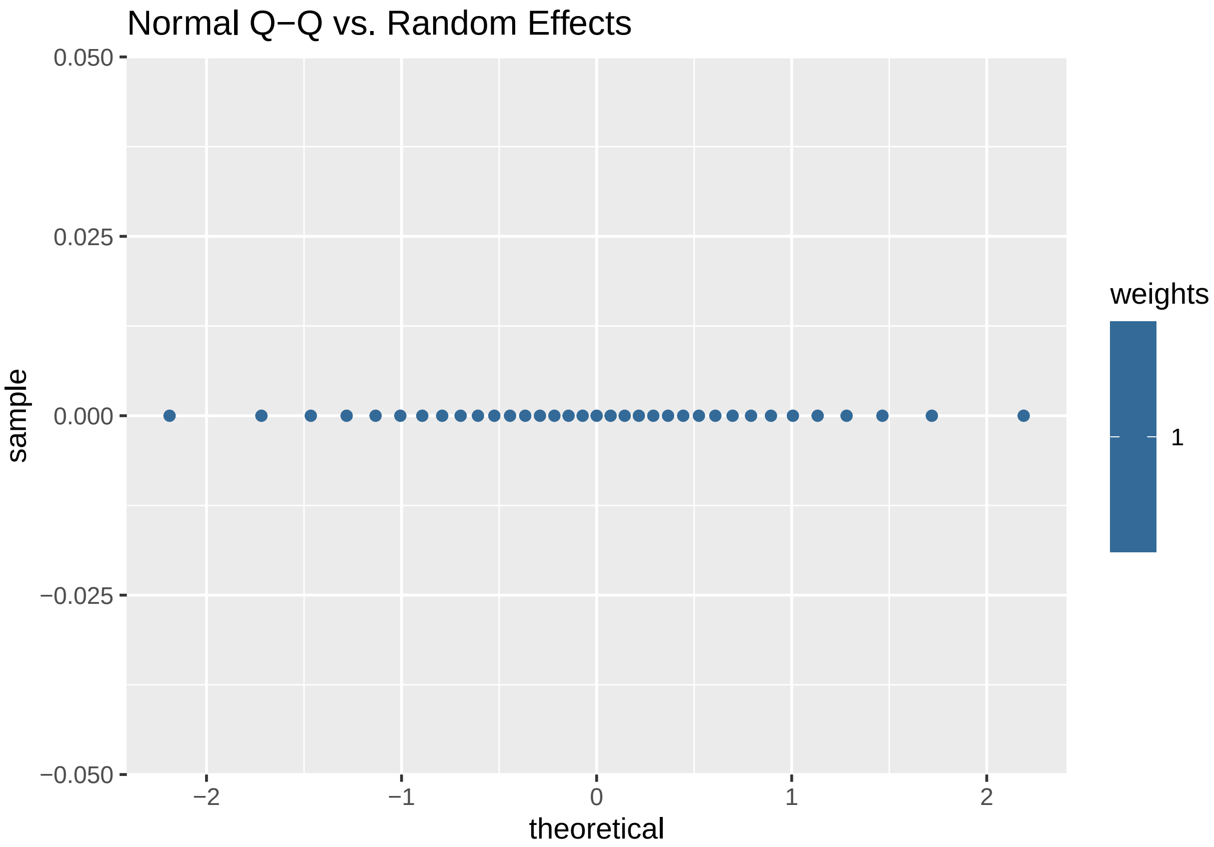
Normal Q−Q vs. Random Effects
0.050
0.025
0.000
−0.025
−0.050
sample
−2
−1
0
1
2
theoretical
weights
1

2.3.6 SST

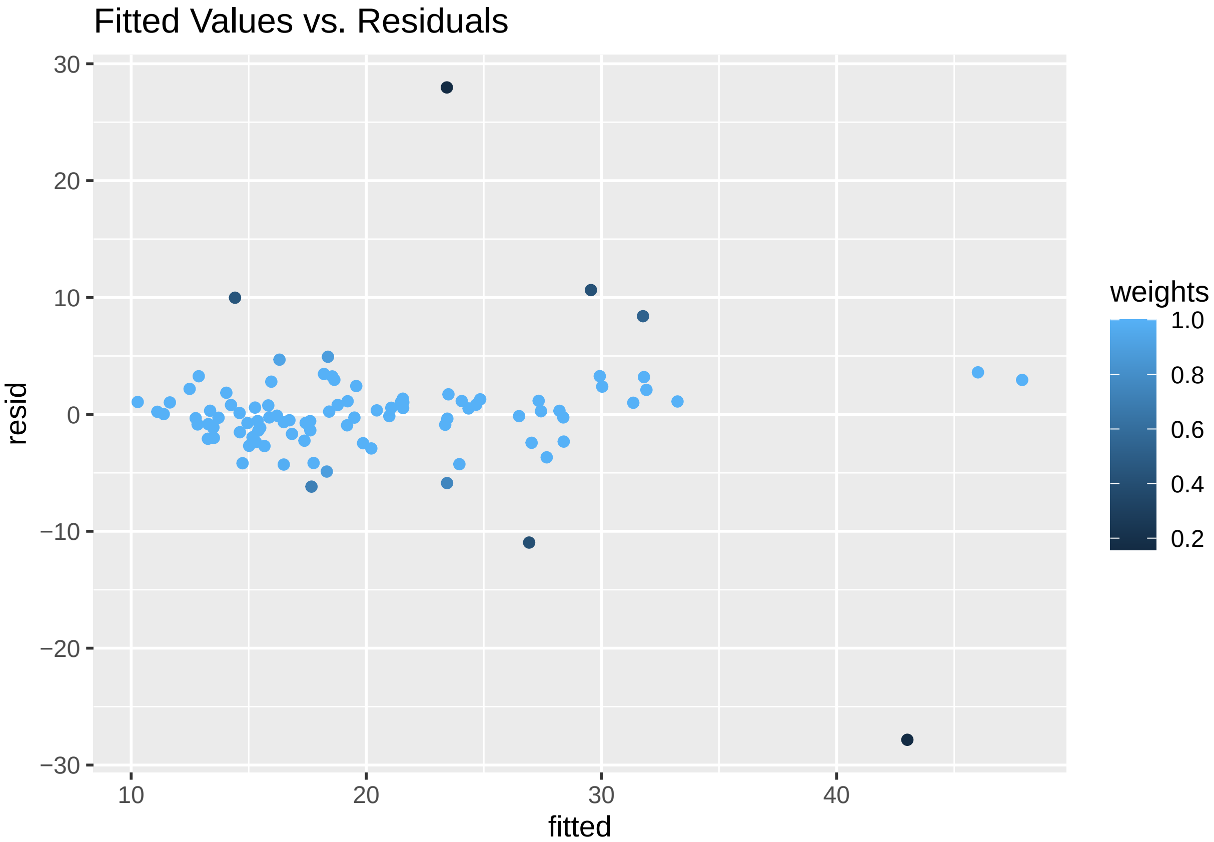

Fitted Values vs. Residuals
30
20
10
0
−10
−20
−30
resid
10
20
30
40
fitted
weights
1.0
0.8
0.6
0.4
0.2

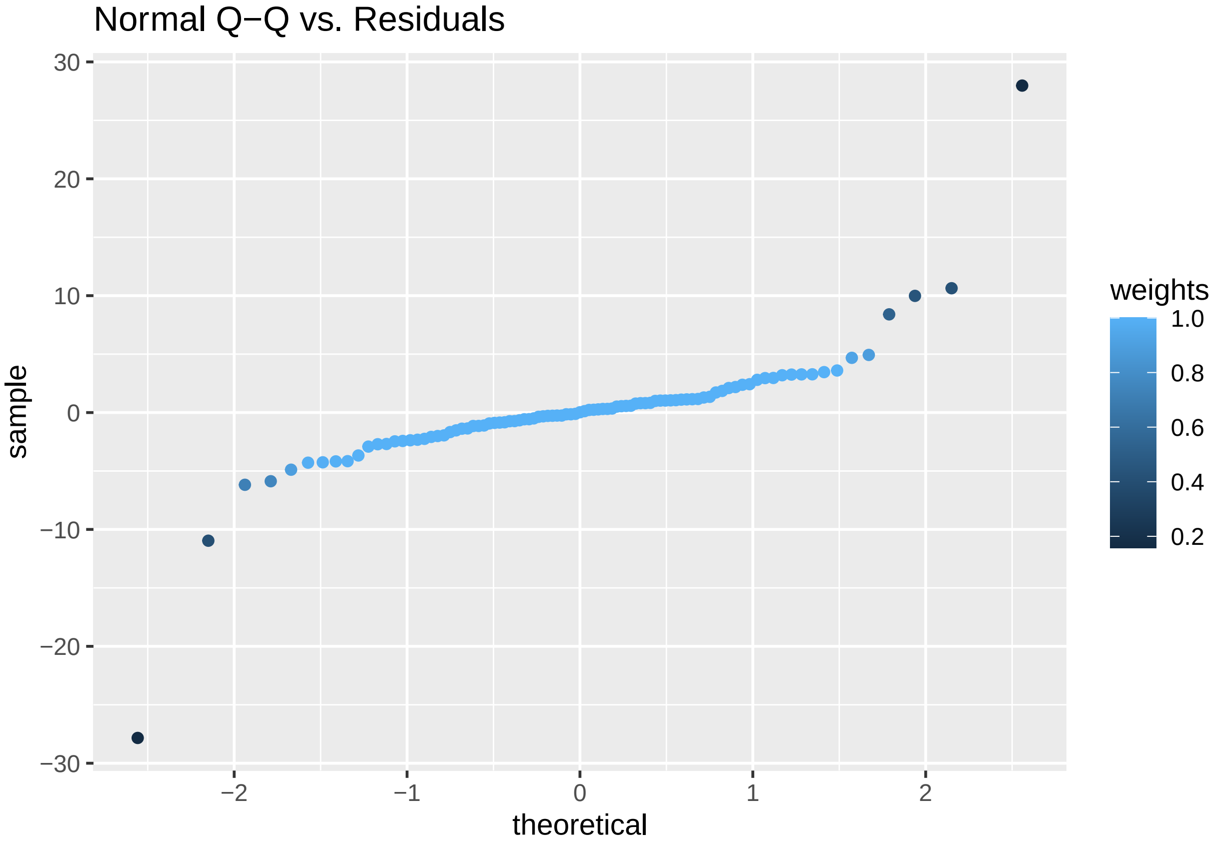

Normal Q−Q vs. Residuals
30
20
10
0
−10
−20
−30
sample
−2
−1
0
1
2
theoretical
weights
1.0
0.8
0.6
0.4
0.2

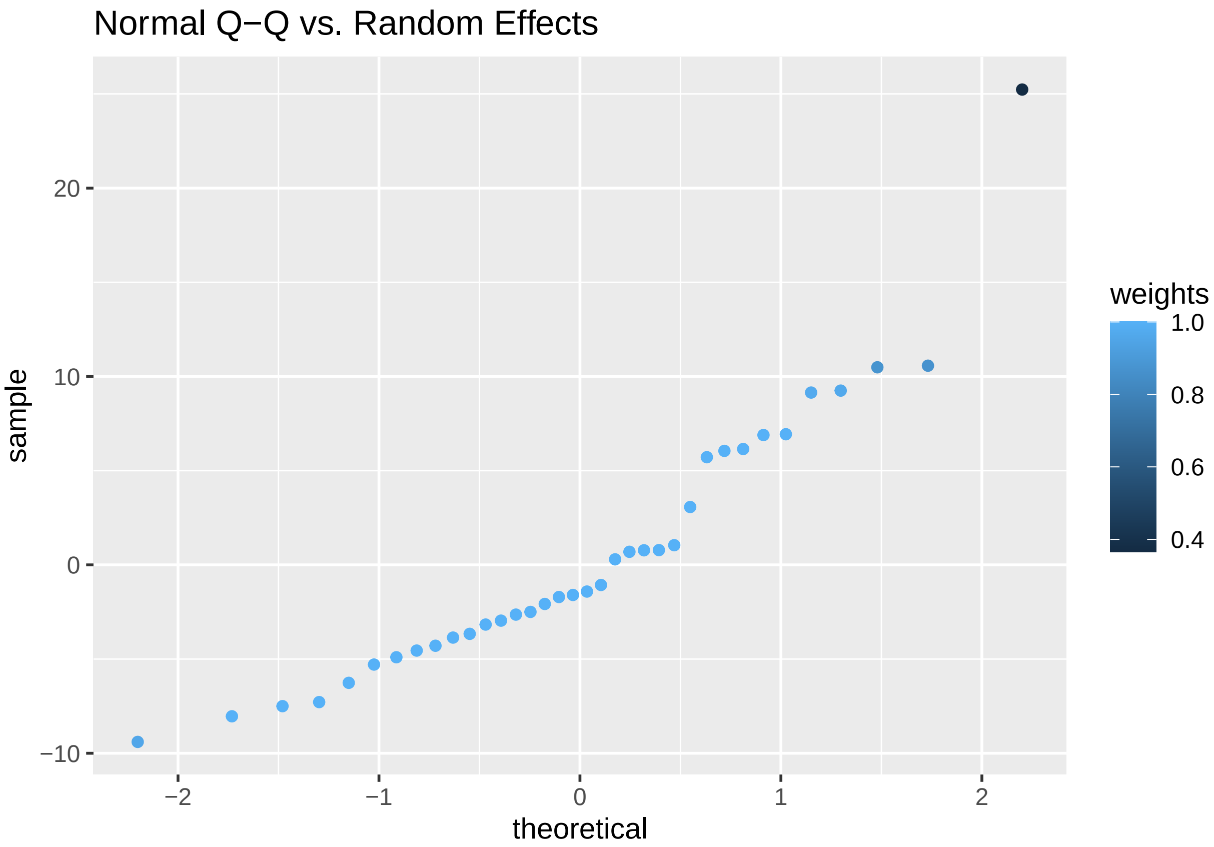
Normal Q−Q vs. Random Effects
sample
theoretical
20
10
0
−10
−2
−1
0
1
2
weights
1.0
0.8
0.6
0.4

3 Results

3.1 Fugl-Meyer Assessment Scale

Group	Time	Baseline	Mean	SE	95% CI lower	95% CI upper	df	T-value	P-value
Chiropractic + PT	At 4-weeks	40.9	64.1	2.0	60.1	68.2	75.7	11.4	0
PT	At 4-weeks	40.9	58.0	2.1	53.9	62.1	75.7	8.2	0
Chiropractic + PT	At 8-weeks	40.9	64.3	2.4	59.6	69.1	86.4	9.8	0
PT	At 8-weeks	40.9	59.8	2.4	55.1	64.6	85.9	7.9	0

Contrast	Time	Mean	SE	95% CI lower	95% CI upper	df	T-value	P-value
Chiropractic + PT - PT	At 4-weeks	6.1	2.9	0.4	11.9	75.6	2.1	0.03767
Chiropractic + PT - PT	At 8-weeks	4.5	3.4	-2.2	11.2	86.2	1.3	0.18664

3.2 Fugl-Meyer Assessment Scale – Upper Extremity

Group	Time	Baseline	Mean	SE	95% CI lower	95% CI upper	df	T-value	P-value
Chiropractic + PT	At 4-weeks	24.1	38.9	1.7	35.5	42.4	79.7	8.6	0
PT	At 4-weeks	24.1	36.0	1.8	32.5	39.5	79.6	6.7	0
Chiropractic + PT	At 8-weeks	24.1	40.3	2.1	36.2	44.4	87.2	7.8	0
PT	At 8-weeks	24.1	37.3	2.1	33.2	41.4	86.9	6.4	0

Contrast	Time	Mean	SE	95% CI lower	95% CI upper	df	T-value	P-value
Chiropractic + PT - PT	At 4-weeks	2.9	2.5	-2.0	7.9	79.6	1.2	0.23550
Chiropractic + PT - PT	At 8-weeks	3.0	2.9	-2.8	8.8	87.0	1.0	0.30678

3.3 Fugl-Meyer Assessment Scale – Lower Extremity

Group	Time	Baseline	Mean	SE	95% CI lower	95% CI upper	df	T-value	P-value
Chiropractic + PT	At 4-weeks	16.8	25.0	0.8	23.3	26.7	71.7	9.7	0
PT	At 4-weeks	16.8	22.1	0.9	20.4	23.8	71.7	6.2	0
Chiropractic + PT	At 8-weeks	16.8	24.2	1.0	22.3	26.2	85.2	7.6	0
PT	At 8-weeks	16.8	22.4	1.0	20.4	24.3	84.4	5.7	0

Contrast	Time	Mean	SE	95% CI lower	95% CI upper	df	T-value	P-value
Chiropractic + PT - PT	At 4-weeks	2.9	1.2	0.5	5.3	71.7	2.4	0.01743
Chiropractic + PT - PT	At 8-weeks	1.9	1.4	-0.9	4.6	84.9	1.3	0.18484

3.4 QOL

Group	Time	Baseline	Mean	SE	95% CI lower	95% CI upper	df	T-value	P-value
Chiropractic + PT	At 4-weeks	122.1	162.5	7.2	148.1	176.9	62.4	5.6	0
PT	At 4-weeks	122.1	171.7	7.4	157.0	186.5	62.3	6.7	0
Chiropractic + PT	At 8-weeks	122.1	190.6	8.1	174.5	206.8	79.7	8.4	0
PT	At 8-weeks	122.1	174.0	8.1	157.9	190.0	76.0	6.4	0

Contrast	Time	Mean	SE	95% CI lower	95% CI upper	df	T-value	P-value
Chiropractic + PT - PT	At 4-weeks	-9.2	10.4	-30.0	11.5	62.2	-0.9	0.37694
Chiropractic + PT - PT	At 8-weeks	16.7	11.5	-6.2	39.6	77.8	1.4	0.15150

3.5 TUG

Group	Time	Baseline	Mean	SE	95% CI lower	95% CI upper	Z-value	P-value
Chiropractic + PT	At 4-weeks	23.7	20.5	1.6	17.4	23.6	-2.0	0.04745
PT	At 4-weeks	23.7	21.7	1.5	18.6	24.7	-1.3	0.19460
Chiropractic + PT	At 8-weeks	23.7	21.3	1.8	17.7	24.9	-1.3	0.19559
PT	At 8-weeks	23.7	20.4	2.0	16.5	24.3	-1.7	0.09693

Contrast	Time	Mean	SE	95% CI lower	95% CI upper	Z-value	P-value
Chiropractic + PT - PT	At 4-weeks	-1.2	2.2	-5.5	3.2	-0.5	0.60241
Chiropractic + PT - PT	At 8-weeks	0.9	2.7	-4.4	6.2	0.3	0.73610

3.6 mRS

Group	Time	Baseline	Mean	SE	95% CI lower	95% CI upper	Z-value	P-value
Chiropractic + PT	At 4-weeks	2.8	2.4	0.2	2.0	2.8	-2.6	0.00942
PT	At 4-weeks	2.8	2.4	0.2	2.0	2.8	-2.4	0.01672
Chiropractic + PT	At 8-weeks	2.8	2.2	0.2	1.8	2.6	-3.4	0.00080
PT	At 8-weeks	2.8	2.1	0.2	1.7	2.5	-3.6	0.00028

Contrast	Time	Mean	SE	95% CI lower	95% CI upper	Z-value	P-value
Chiropractic + PT - PT	At 4-weeks	0.0	0.3	-0.5	0.5	-0.1	0.94292
Chiropractic + PT - PT	At 8-weeks	0.1	0.3	-0.4	0.6	0.3	0.74960

3.7 SST

Mean difference (MD) is defined as [Chiropractic + PT − (Chiropractic + PT)Baseline] − [PT − (PT)Baseline].

Group	Time	Mean	SE	95% CI lower	95% CI upper	Z-value	P-value
Chiropractic + PT	Baseline	21.3	2.1	17.2	25.4	NA	NA
PT	Baseline	22.7	2.0	18.7	26.7	NA	NA
Chiropractic + PT	At 4-weeks	17.9	2.1	13.8	22.0	-1.1	0.25733
PT	At 4-weeks	20.8	2.0	16.8	24.8	-0.6	0.51574
Chiropractic + PT	At 8-weeks	17.4	2.3	12.8	21.9	-1.2	0.21269
PT	At 8-weeks	17.8	2.6	12.6	22.9	-1.5	0.14150

Contrast	Time	Mean	SE	95% CI lower	95% CI upper	Z-Value	P-value
MD	At 4-weeks	-1.5	4.2	-9.6	6.6	-0.4	0.71933
MD	At 8-weeks	1.0	4.6	-8.0	9.9	0.2	0.83004
